# Supplementary material for: Directed Lithiation of Protected 4-Chloropyrrolopyrimidine: Addition to Aldehydes and Ketones Aided by Bis(2-dimethylaminoethyl)ether
Source: Molecules. 2023 Jan 17;28(3):932. doi: 10.3390/molecules28030932 (PMC9919650; doi:10.3390/molecules28030932)
Supplement: Supplementary file 1 [file molecules-28-00932-s001.zip › molecules-2138484-supplementary.pdf]

Supporting information

Directed lithiation of protected 4-chloropyrrolopyrimidine: addition to aldehydes and ketones aided by bis(2-dimethylaminoethyl)ether

Frithjof Bjørnstad, Eirik Sundby, Bård Helge Hoff

Contents

NMR spectra of the products 3a-p 2

NMR spectra of compounds 1 and 4-8 18

NMR spectra of the products 3a-p

Compound 3a

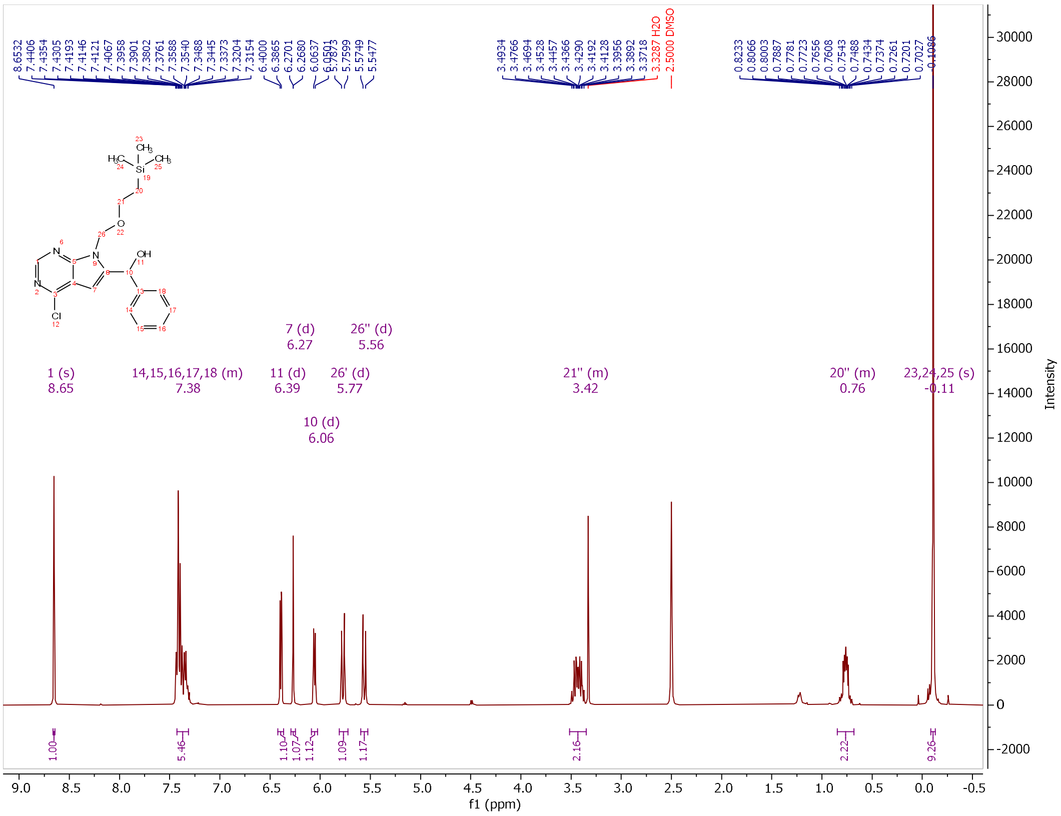

Figure S1. ^{1}H NMR (400 MHz, DMSO-d_6) spectrum of compound **3a**.

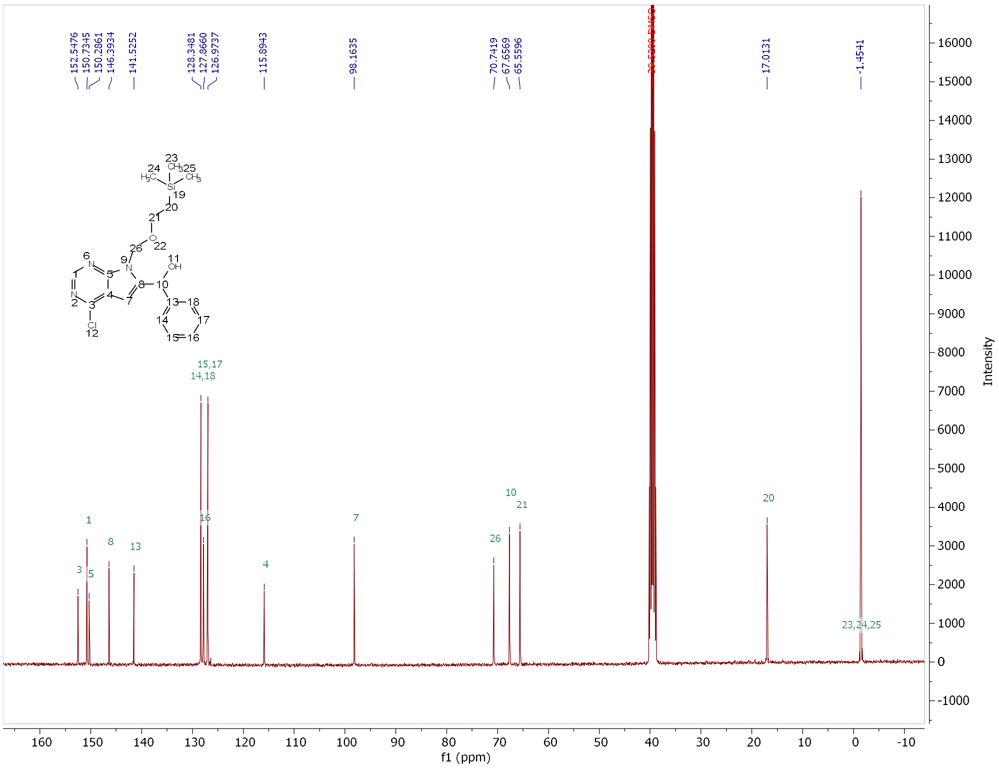

Figure S2. ^{13}C NMR (100 MHz, DMSO-d_6) spectrum of compound **3a**.

Compound 3b

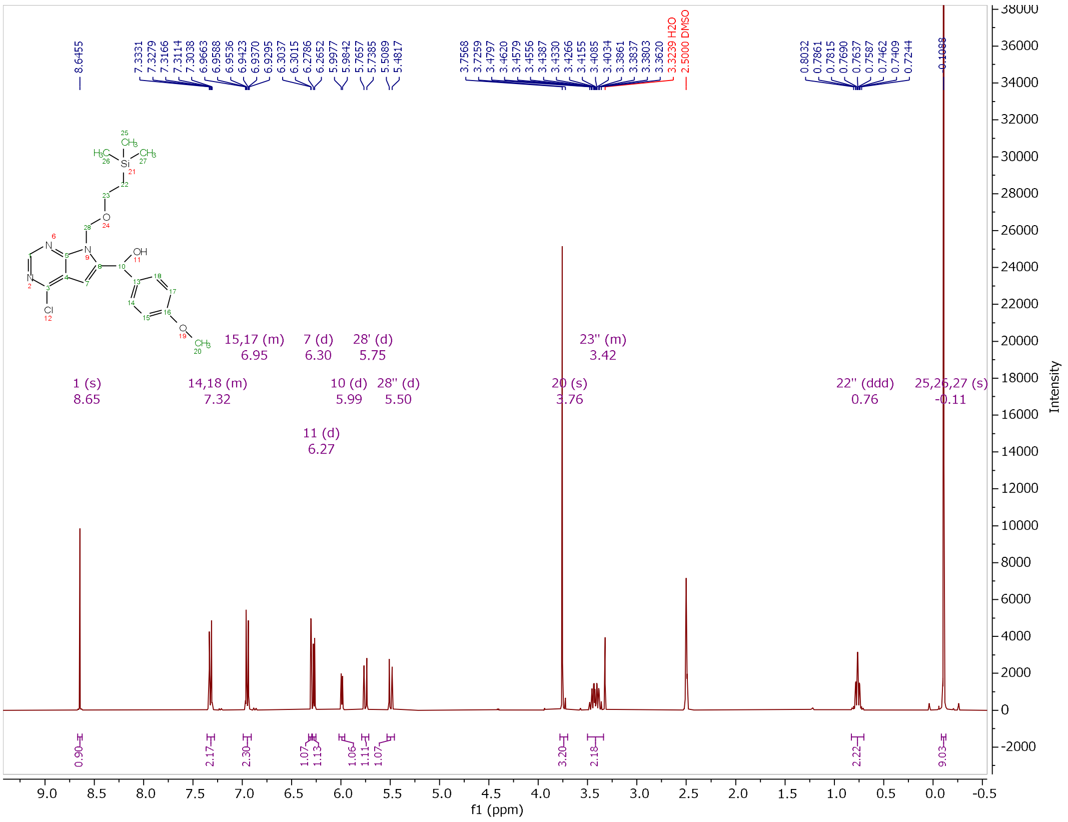

Figure S3. ^{1}H NMR (600 MHz, DMSO-d_6) spectrum of compound **3b**.

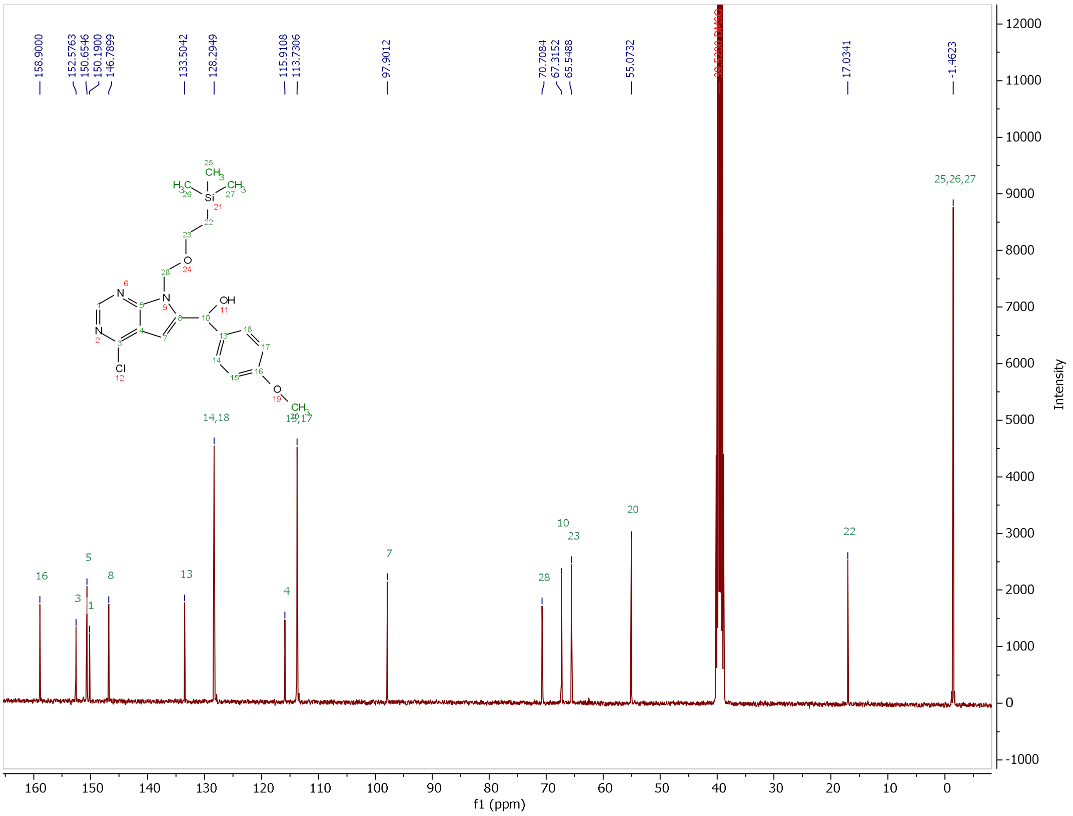

Figure S4. ^{13}C NMR (150 MHz, DMSO-d_6) spectrum of compound **3b**.

Compound 3c

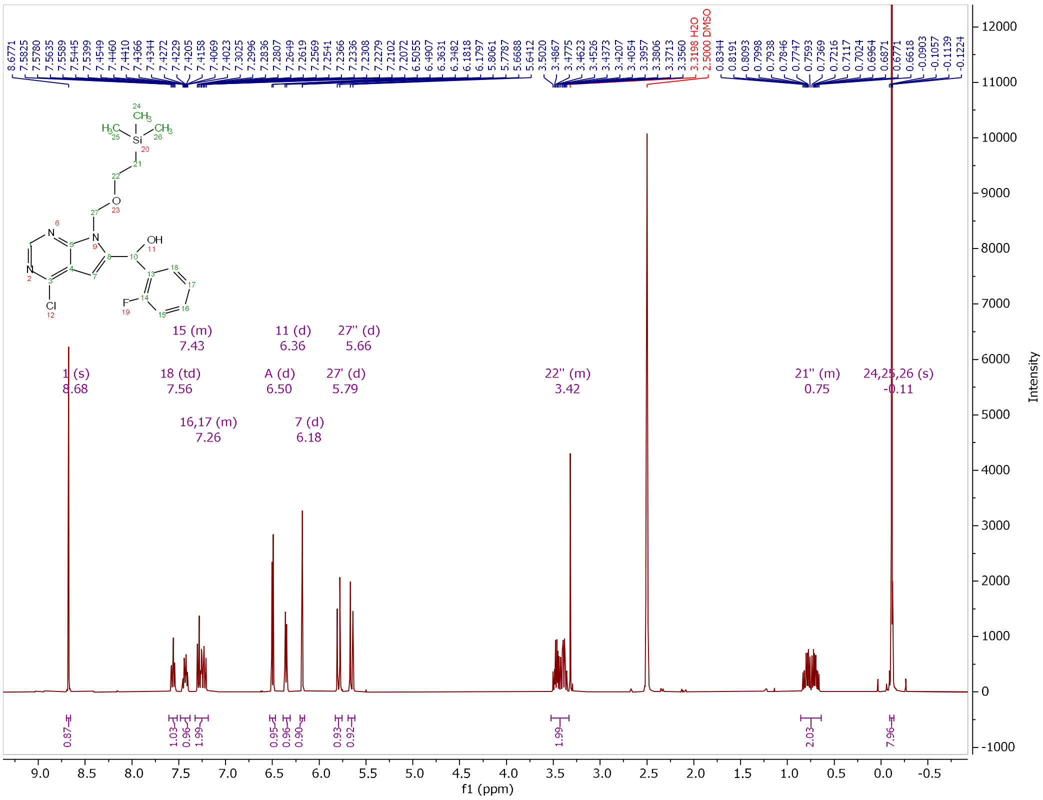

Figure S5. ^{1}H NMR (400 MHz, DMSO-d_6) spectrum of compound **3c**.

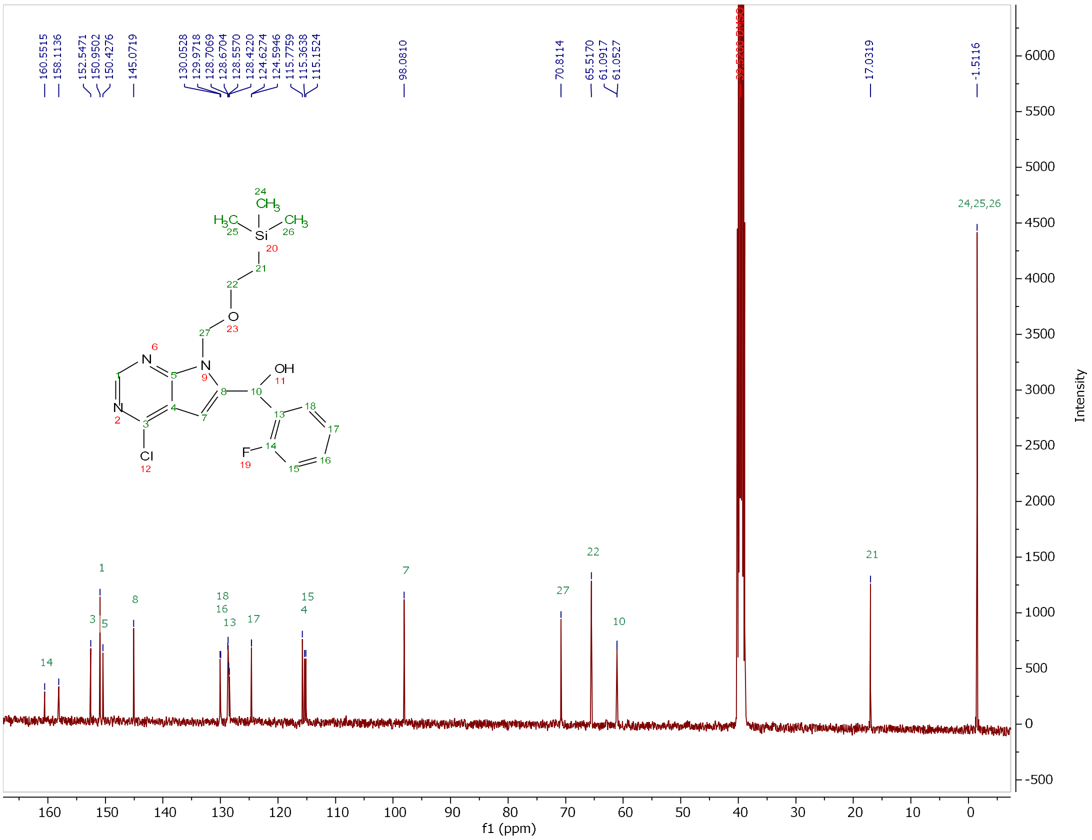

Figure S6. ^{13}C NMR (100 MHz, DMSO-d_6) spectrum of compound **3c**.

Compound 3d

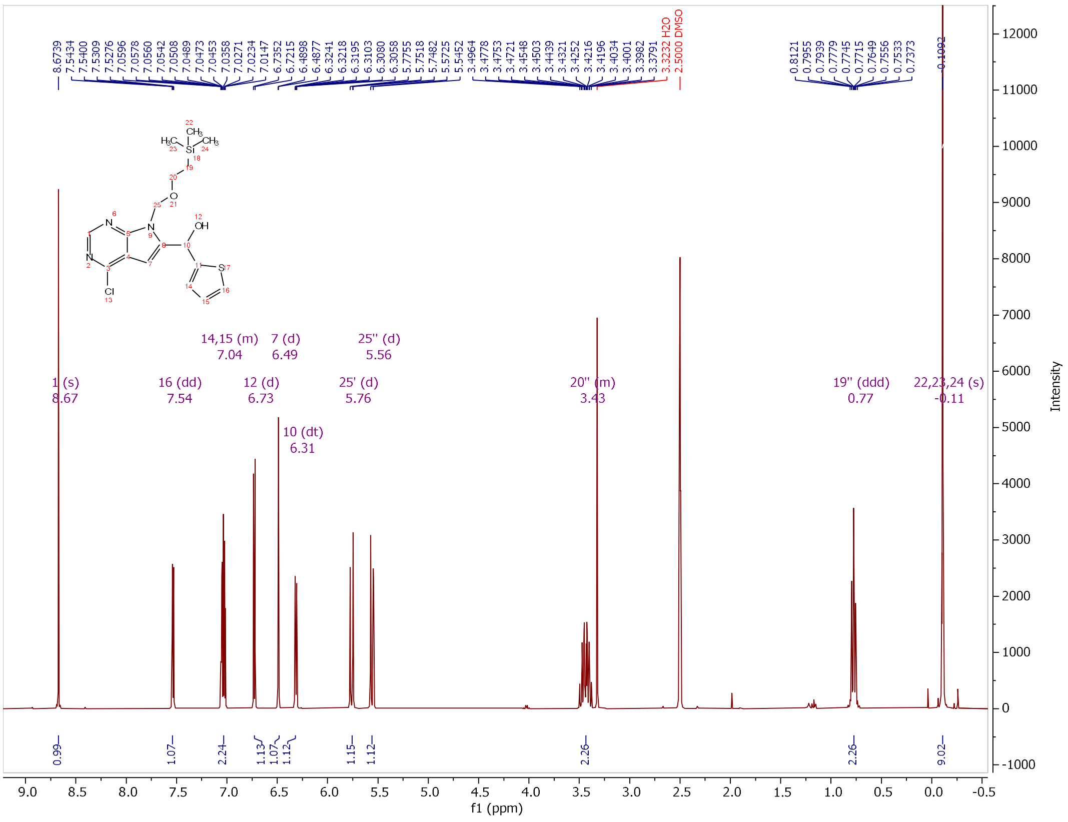

Figure S7. ^{1}H NMR (400 MHz, DMSO-d_6) spectrum of compound **3d**.

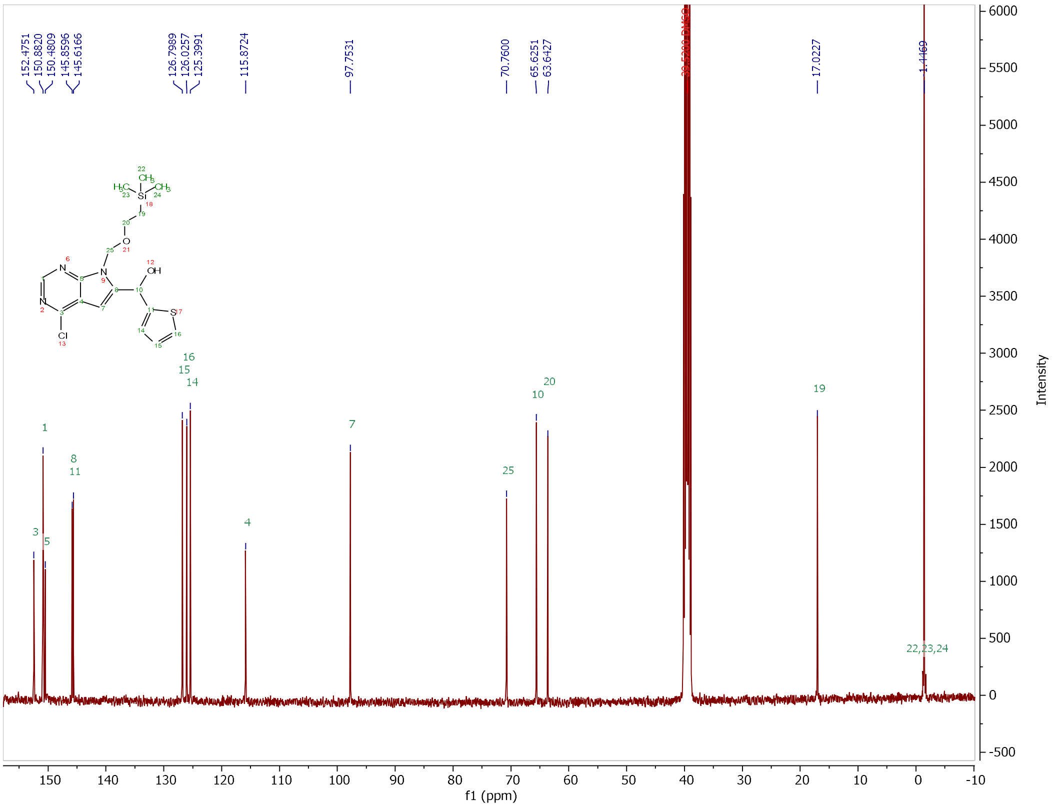

Figure S8. ^{13}C NMR (100 MHz, DMSO-d_6) spectrum of compound **3d**.

Compound 3e

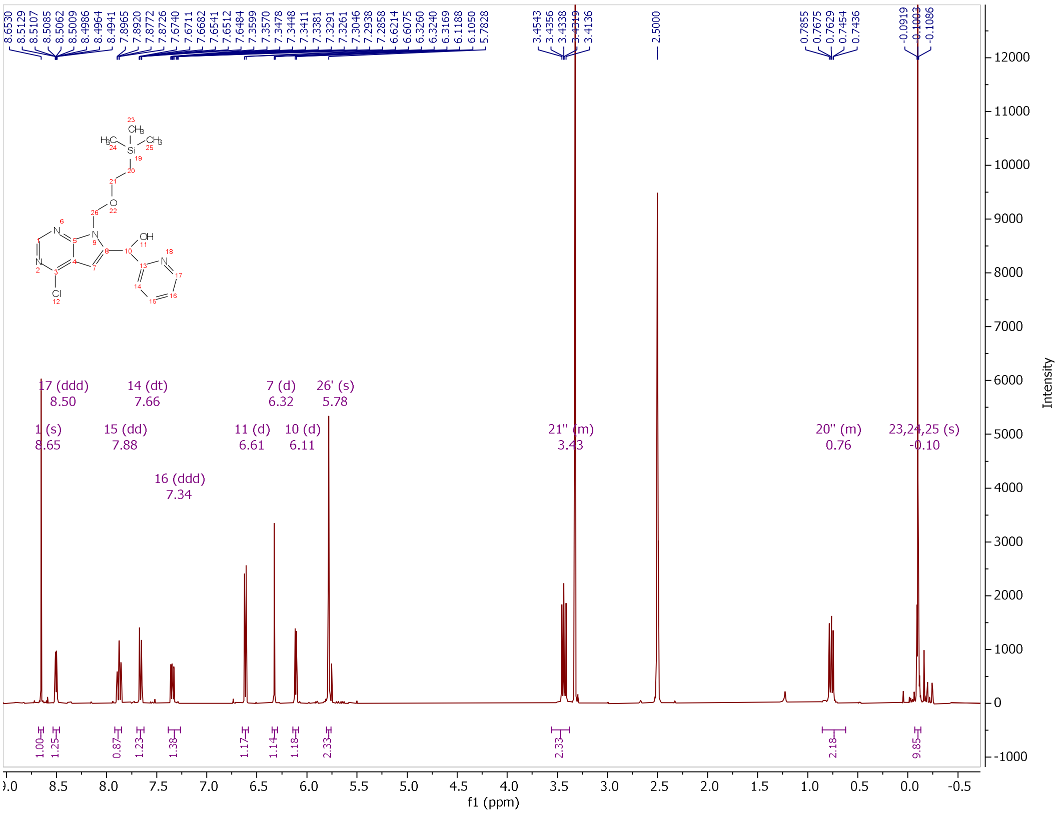

Figure S9. ^{1}H NMR (150 MHz, DMSO-d_6) spectrum of compound **3e**.

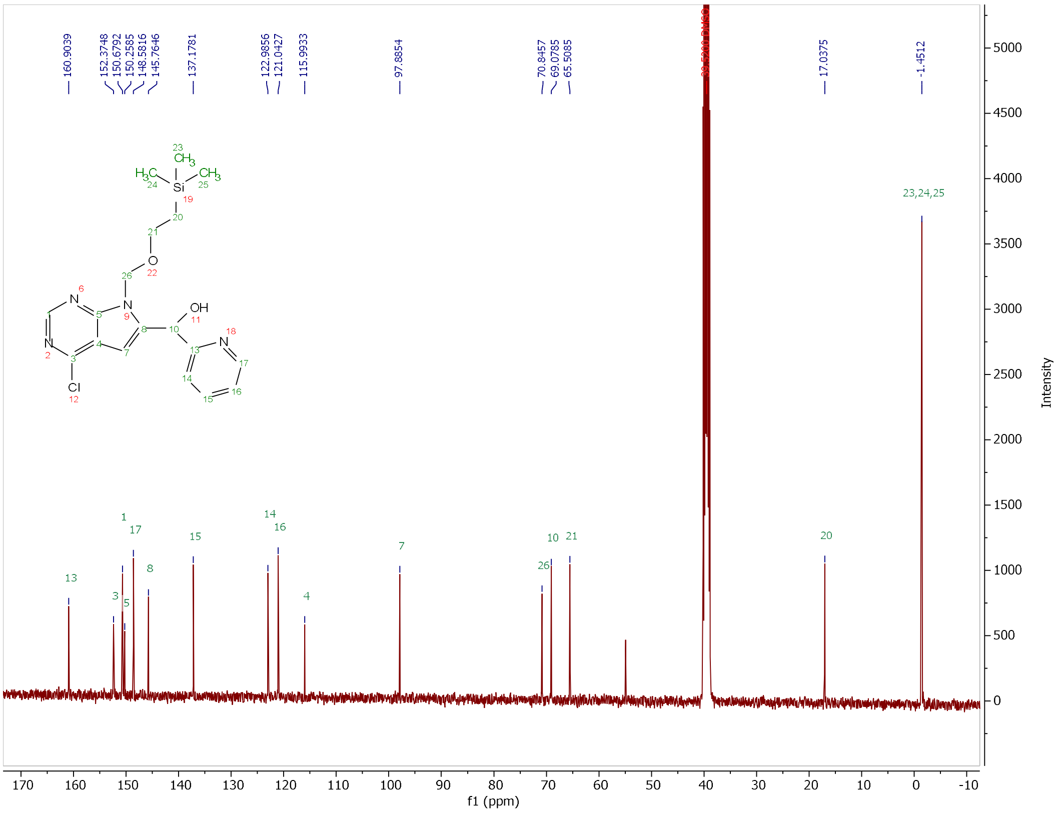

Figure S10. ^{13}C NMR (100 MHz, DMSO-d_6) spectrum of compound **3e**.

Compound 3f

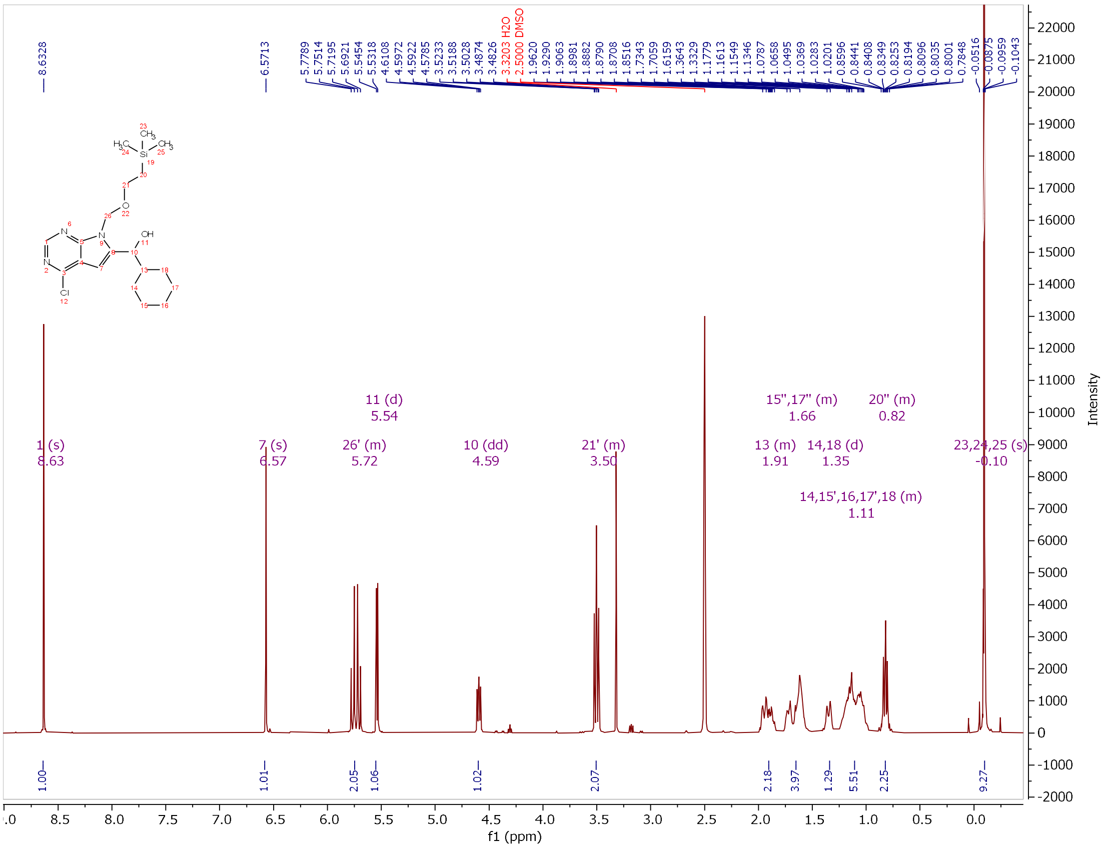

Figure S11. ^{1}H NMR (600 MHz, DMSO-d_6) spectrum of compound **3f**.

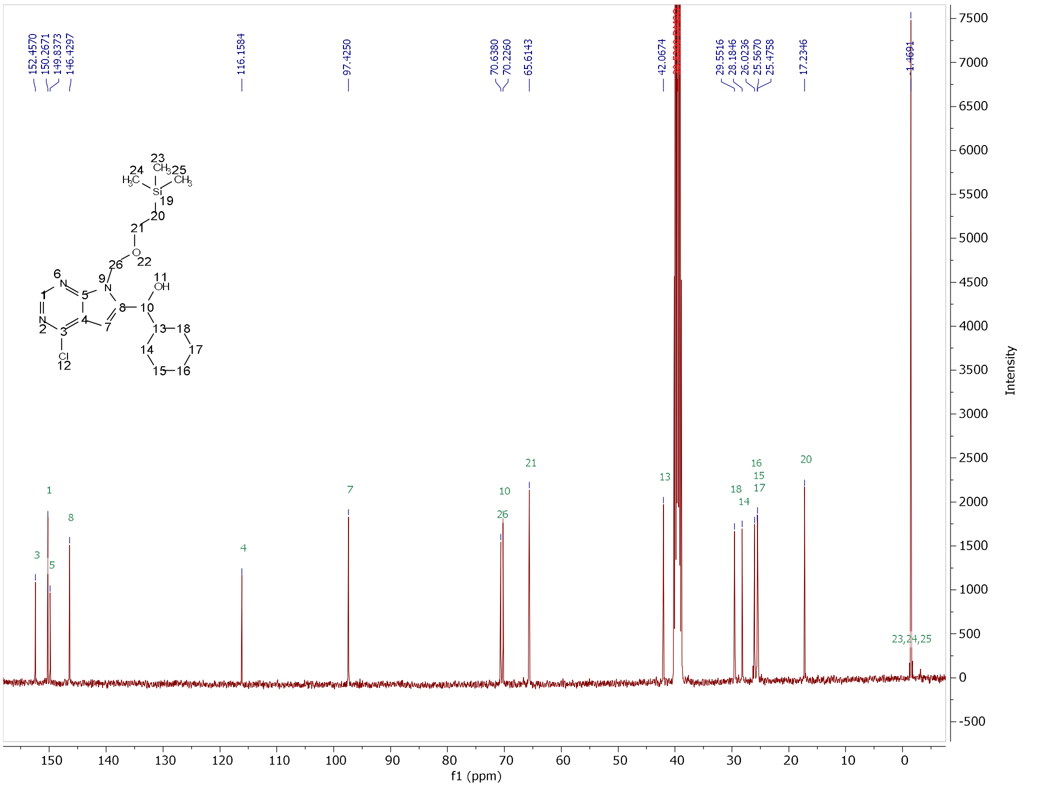

Figure S12. ^{13}C NMR (150 MHz, DMSO-d_6) spectrum of compound **3f**.

Compound 3g

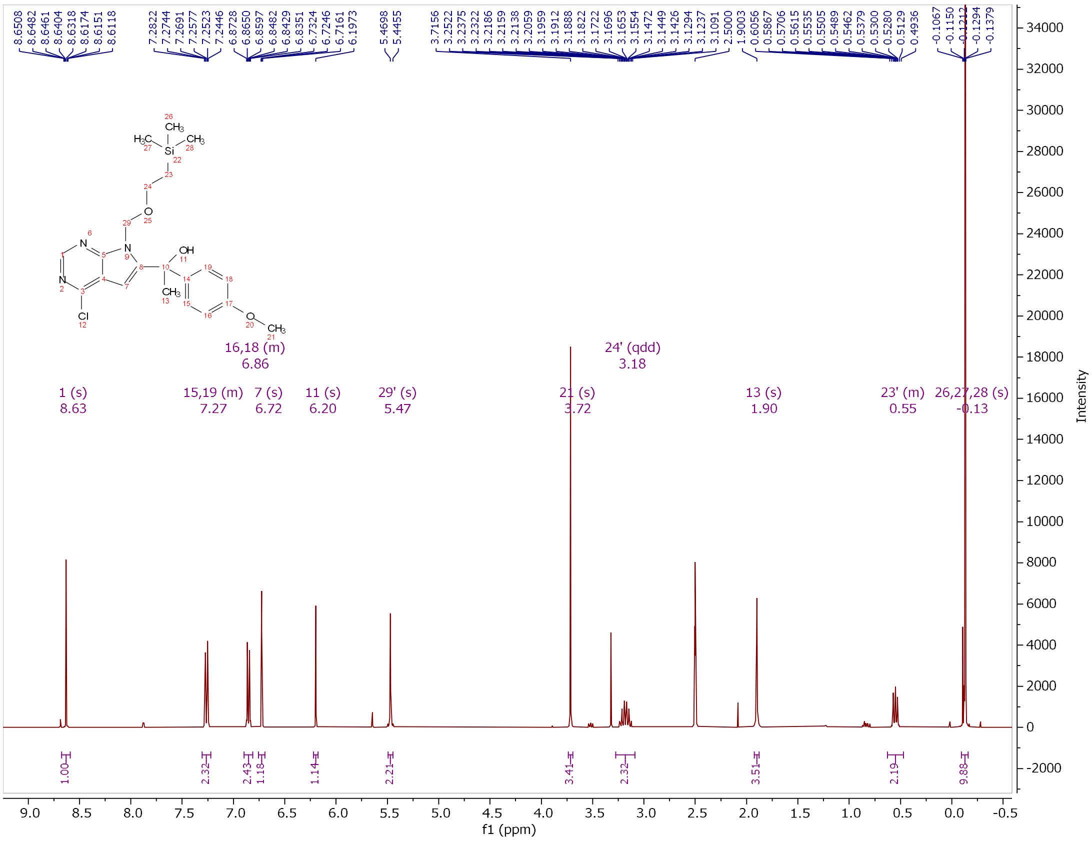

Figure S13. ^{1}H NMR (400 MHz, DMSO-d_6) spectrum of compound **3g**.

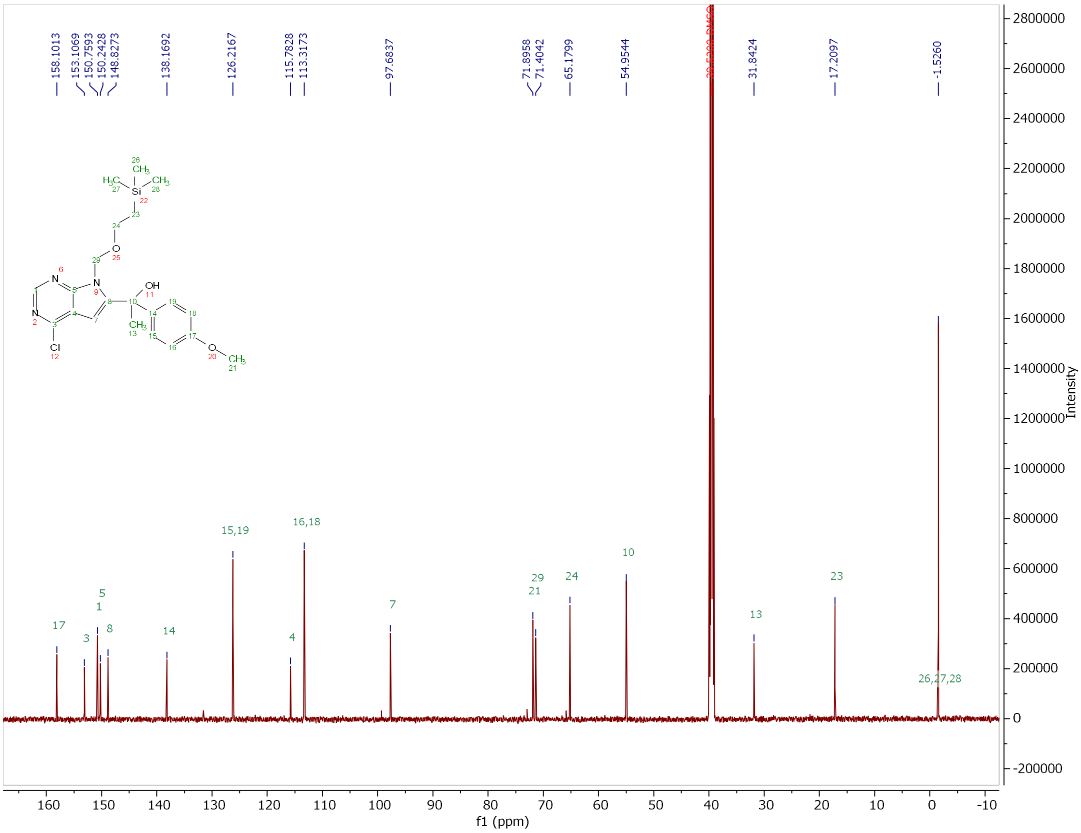

Figure S14. ^{13}C NMR (100 MHz, DMSO-d_6) spectrum of compound **3g**.

Compound 3h

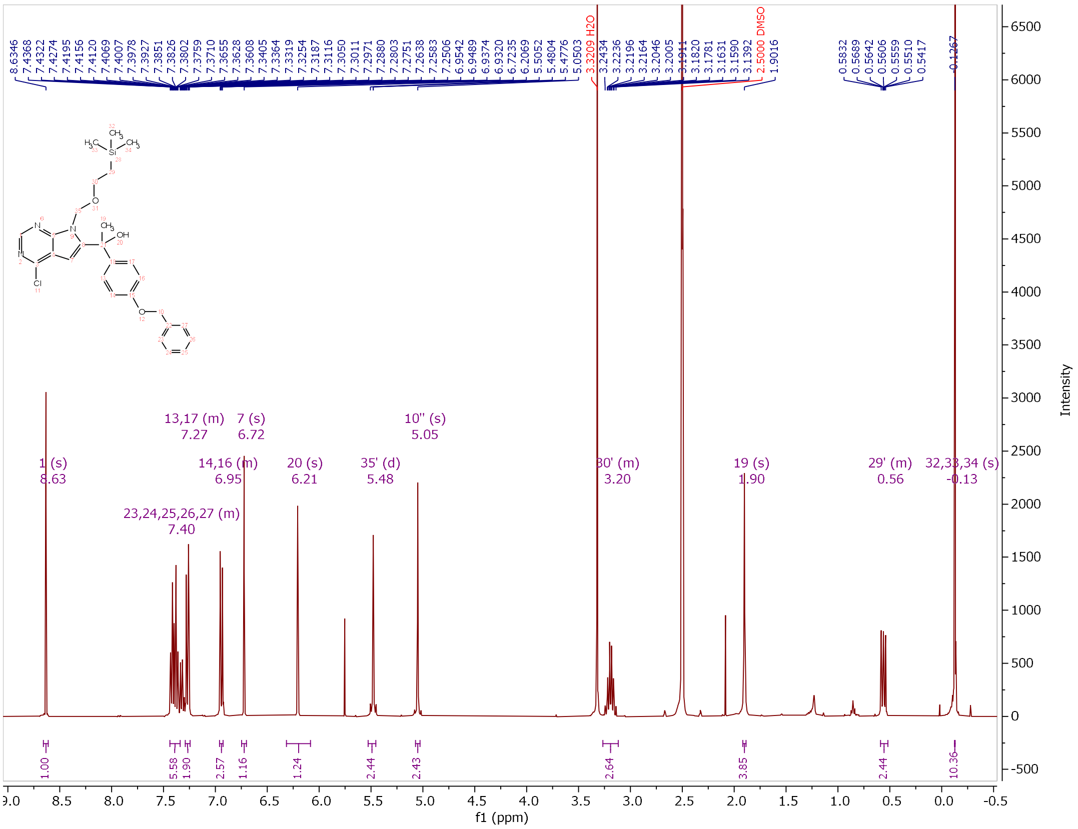

Figure S15. ^{1}H NMR (400 MHz, DMSO-d_6) spectrum of compound **3h**.

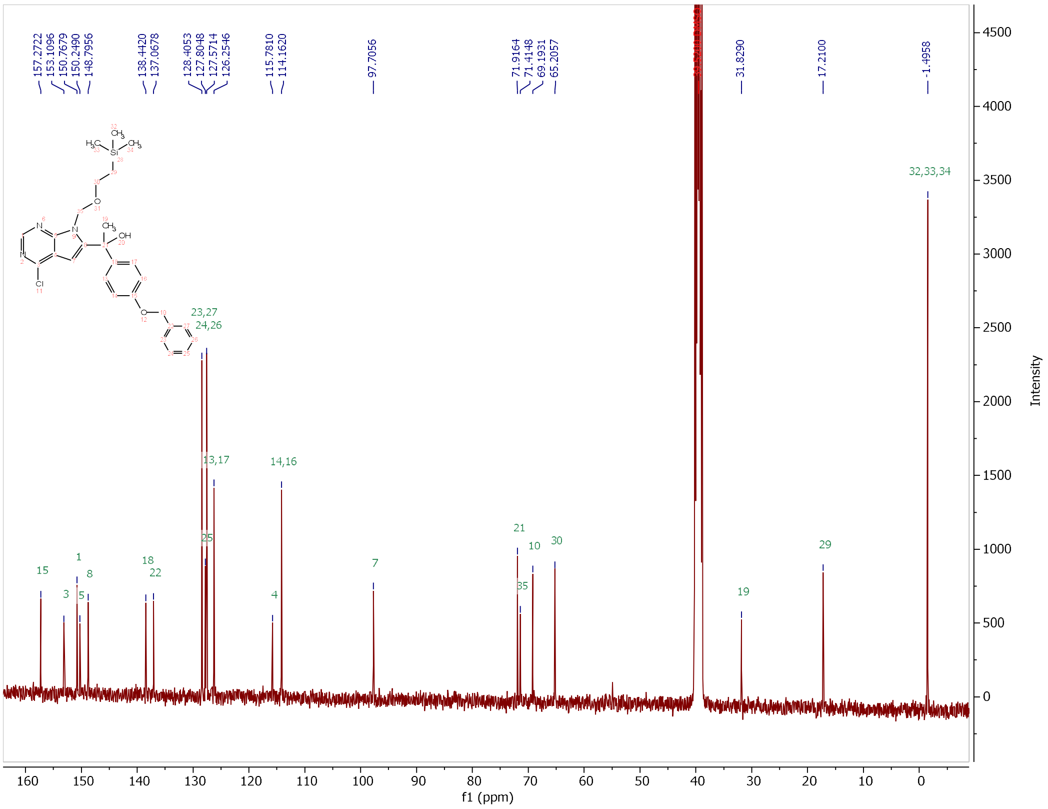

Figure S16. ^{13}C NMR (100 MHz, DMSO-d_6) spectrum of compound **3h**.

Compound 3i

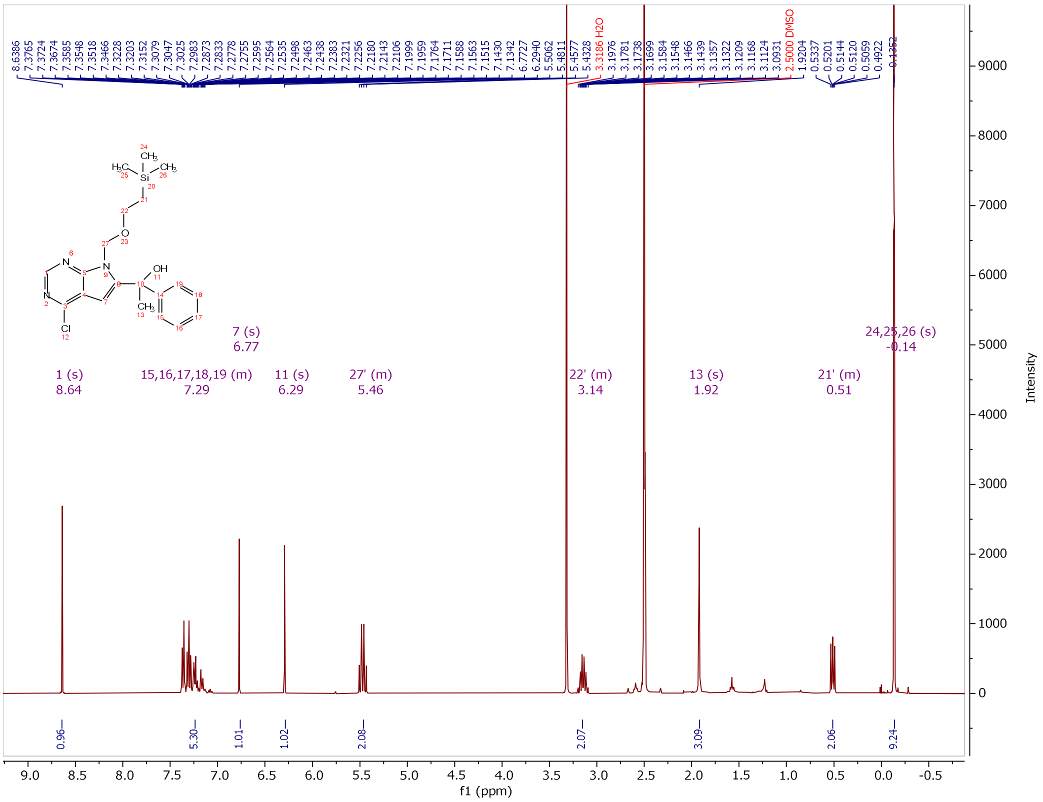

Figure S17. ^{1}H NMR (400 MHz, DMSO-d_6) spectrum of compound **3i**.

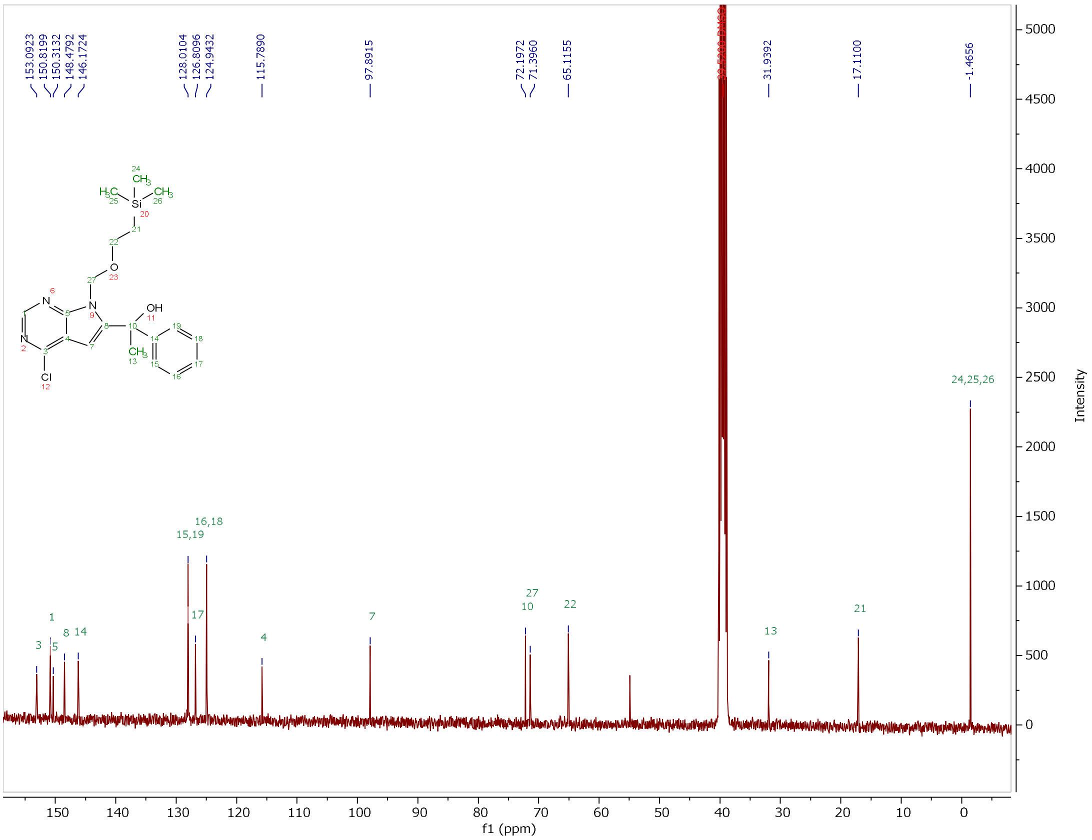

Figure S18. ^{13}C NMR (100 MHz, DMSO-d_6) spectrum of compound **3i**.

Compound 3j

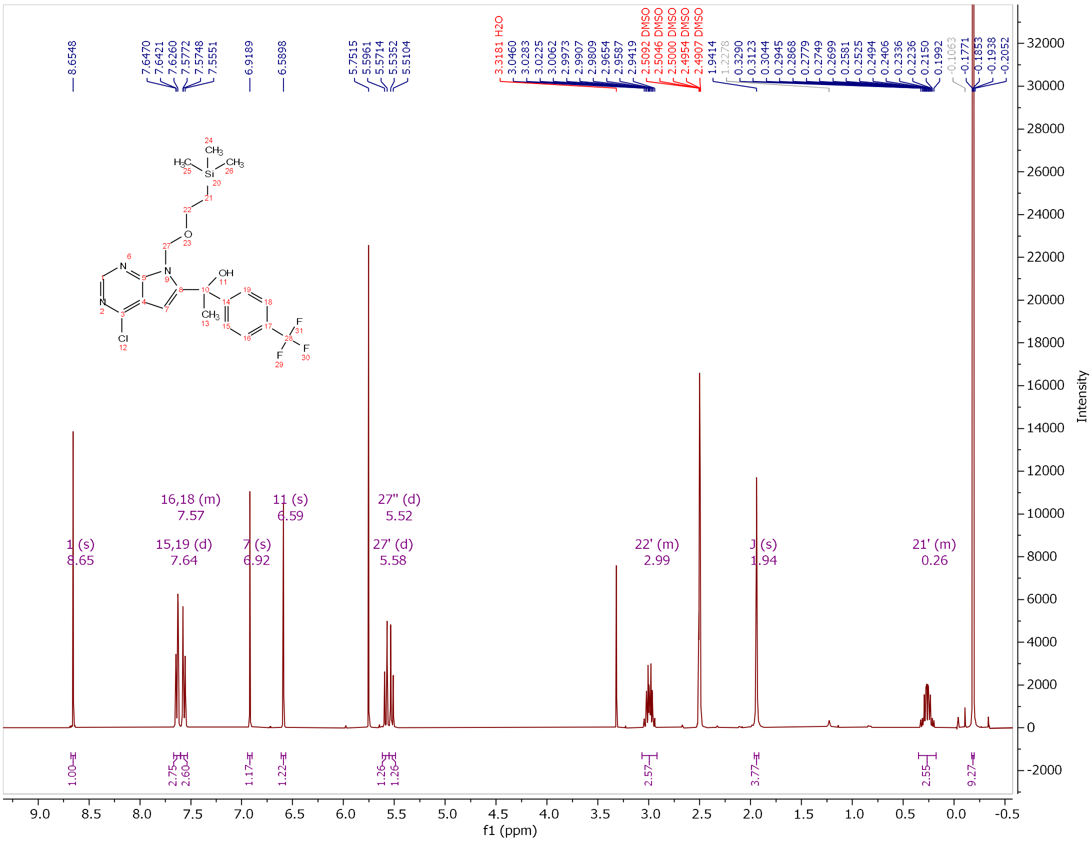

Figure S19. ^{1}H NMR (400 MHz, DMSO-d_6) spectrum of compound **3j**.

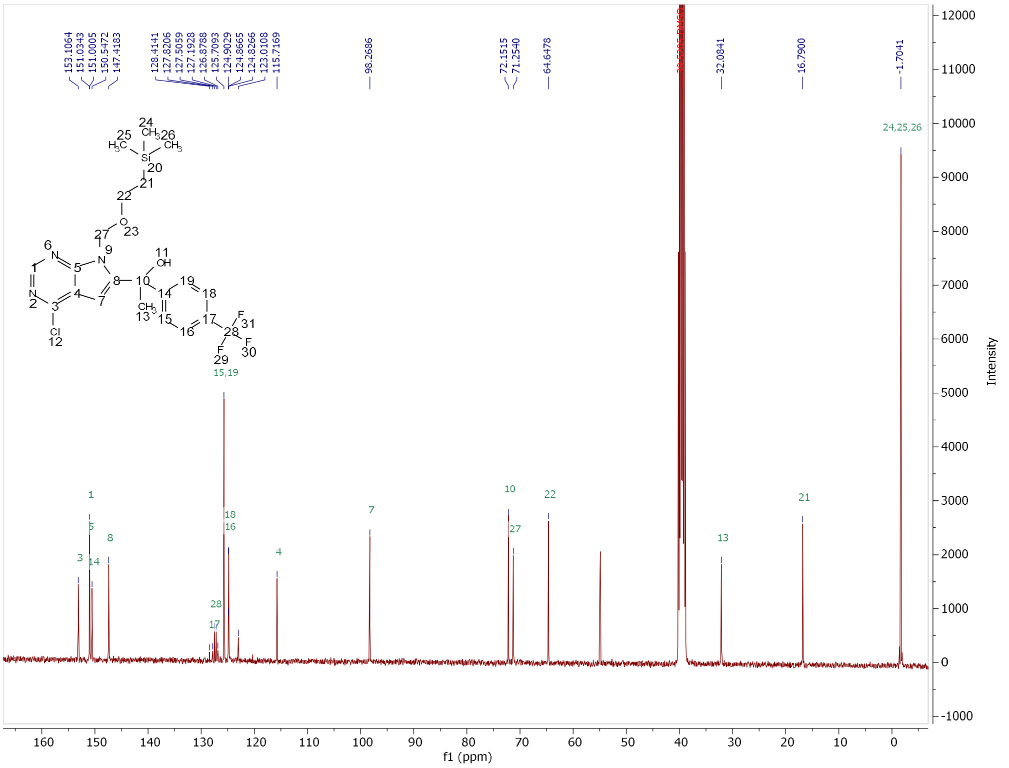

Figure S20. ^{13}C NMR (100 MHz, DMSO-d_6) spectrum of compound **3j**.

Compound 3k

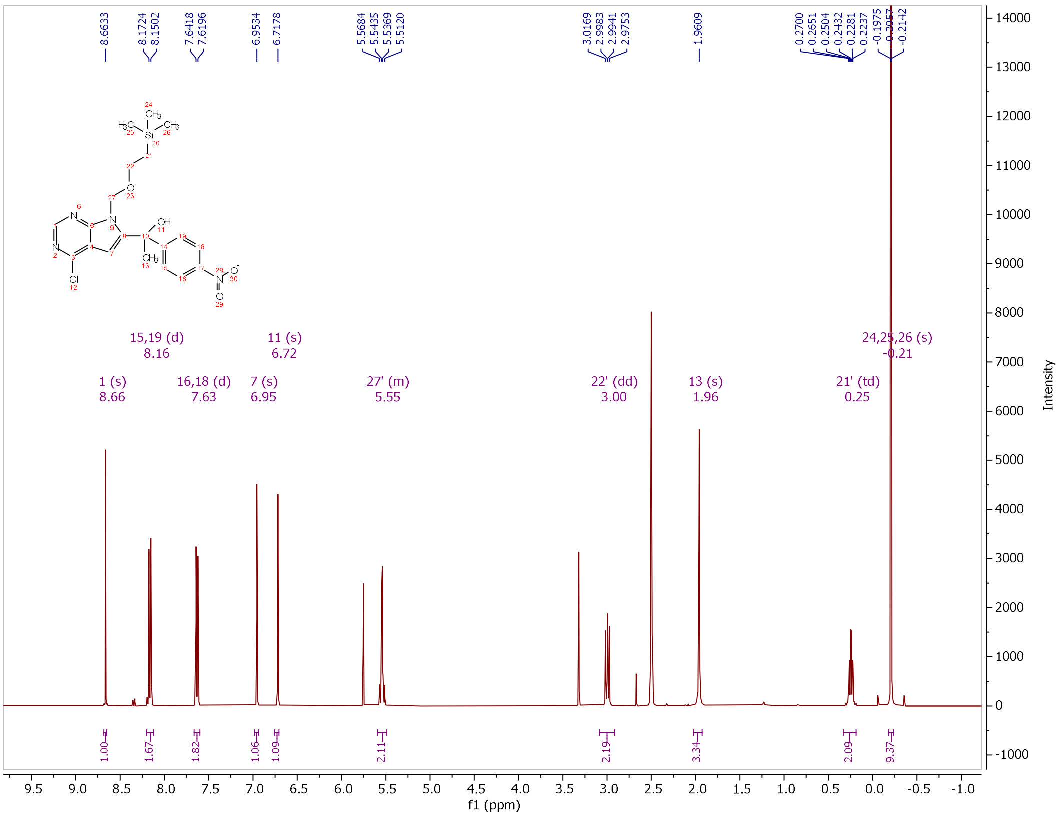

Figure S21. ^{1}H NMR (400 MHz, DMSO-d_6) spectrum of compound **3k**.

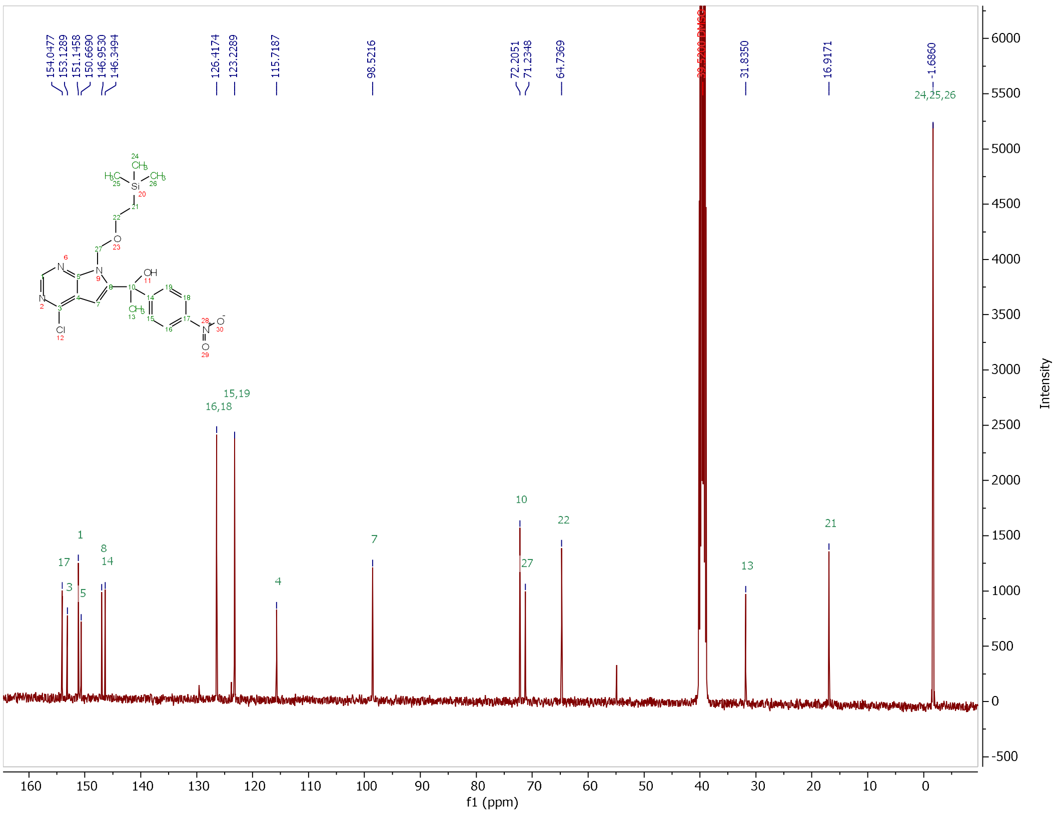

Figure S22. ^{13}C NMR (100 MHz, DMSO-d_6) spectrum of compound **3k**.

Compound 3l

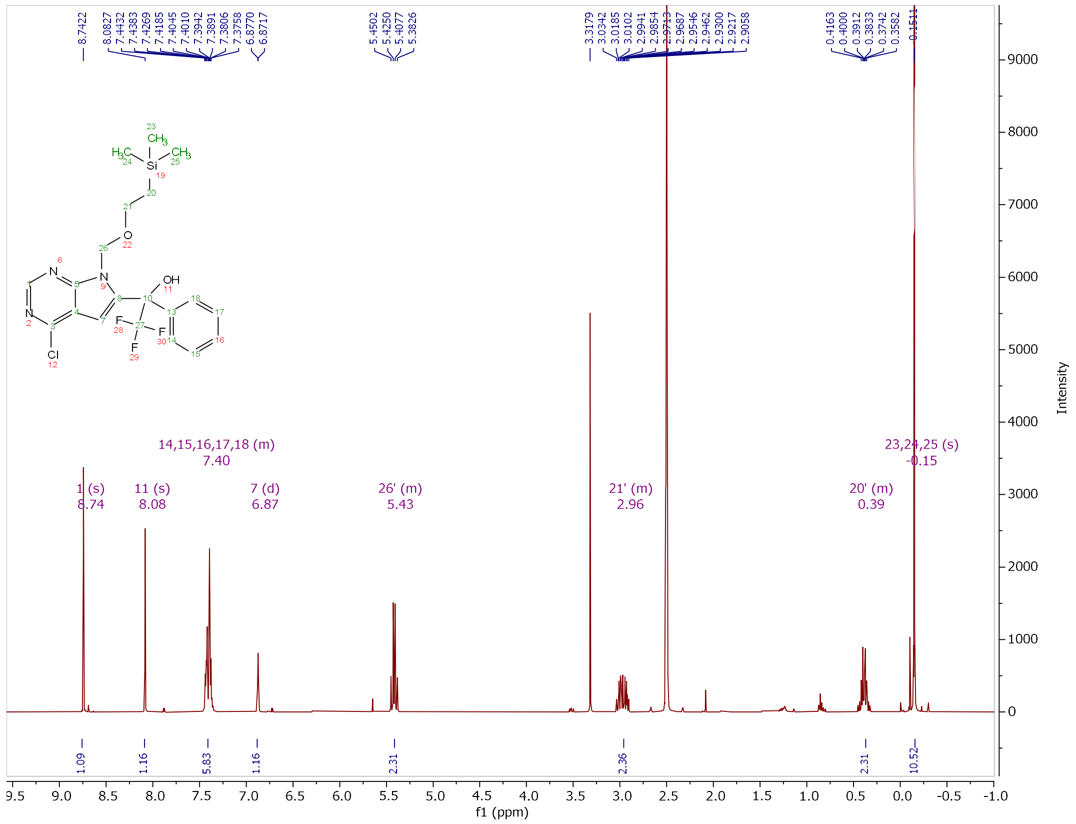

Figure S23. ^{1}H NMR (400 MHz, DMSO-d_6) spectrum of compound **3l**.

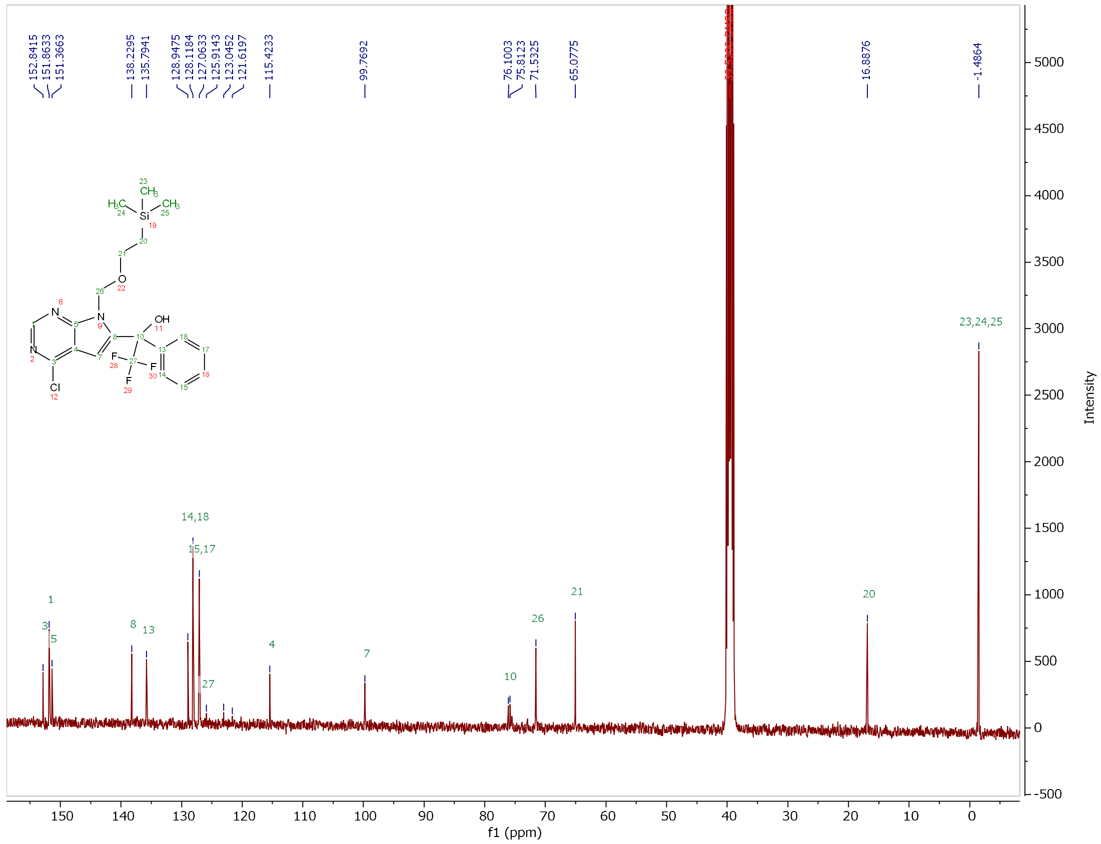

Figure S24. ^{13}C NMR (100 MHz, DMSO-d_6) spectrum of compound **3l**.

Compound 3m

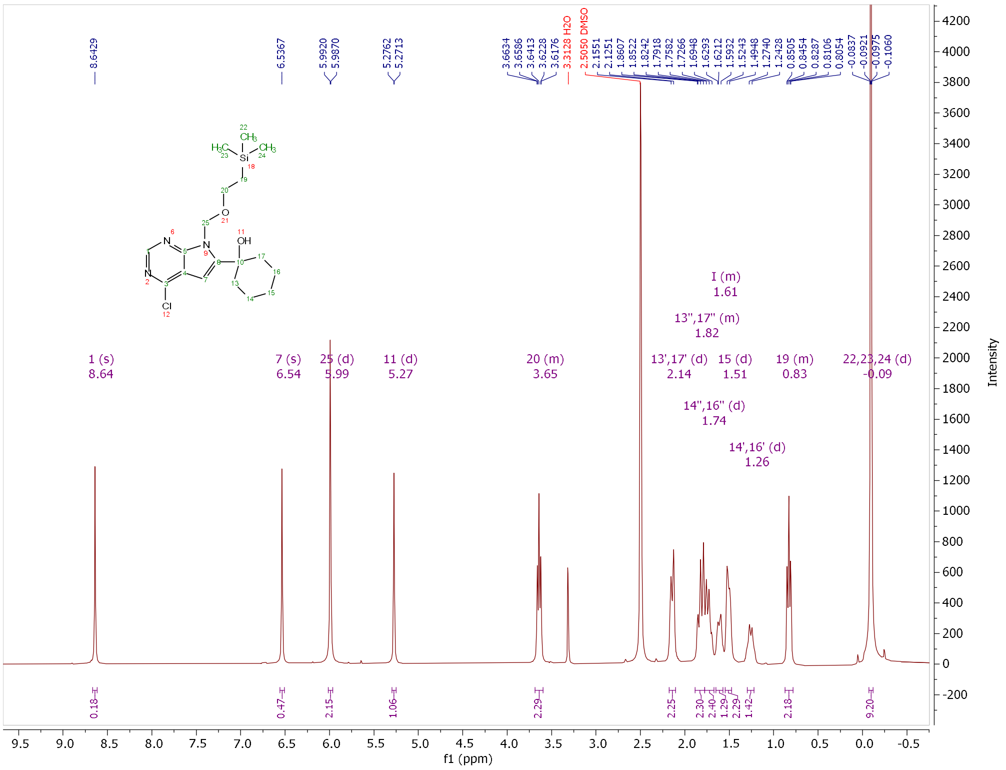

Figure S25. ^{1}H NMR (600 MHz, DMSO-d_6) spectrum of compound **3m**.

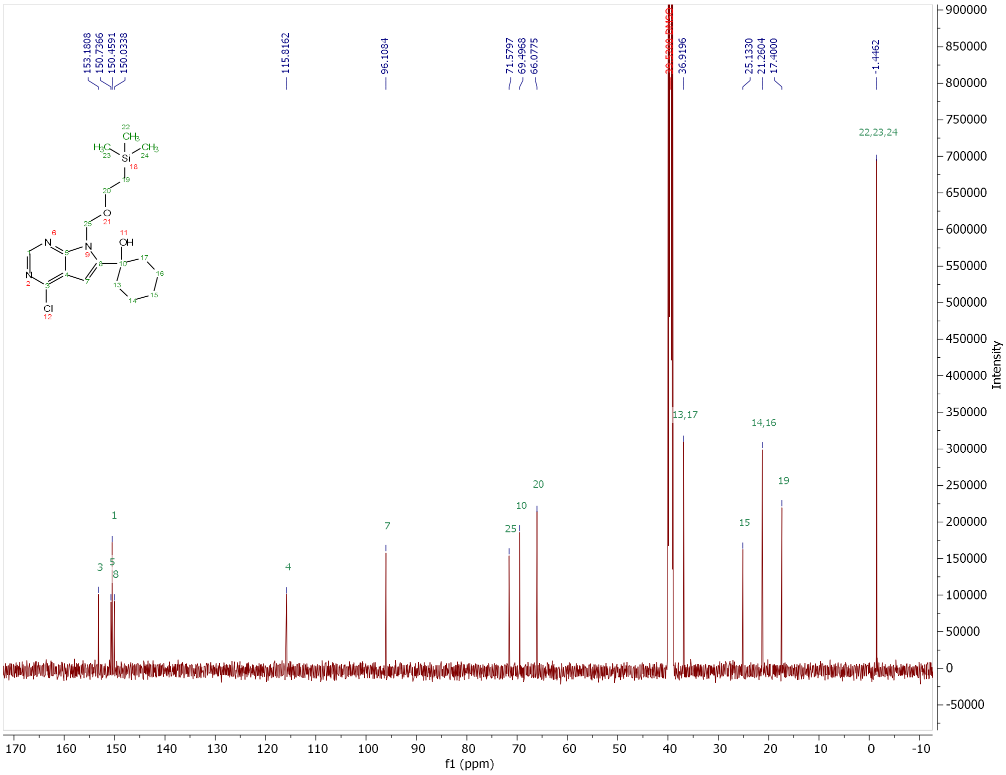

Figure S26. ^{13}C NMR (100 MHz, DMSO-d_6) spectrum of compound **3m**.

Compound 3n

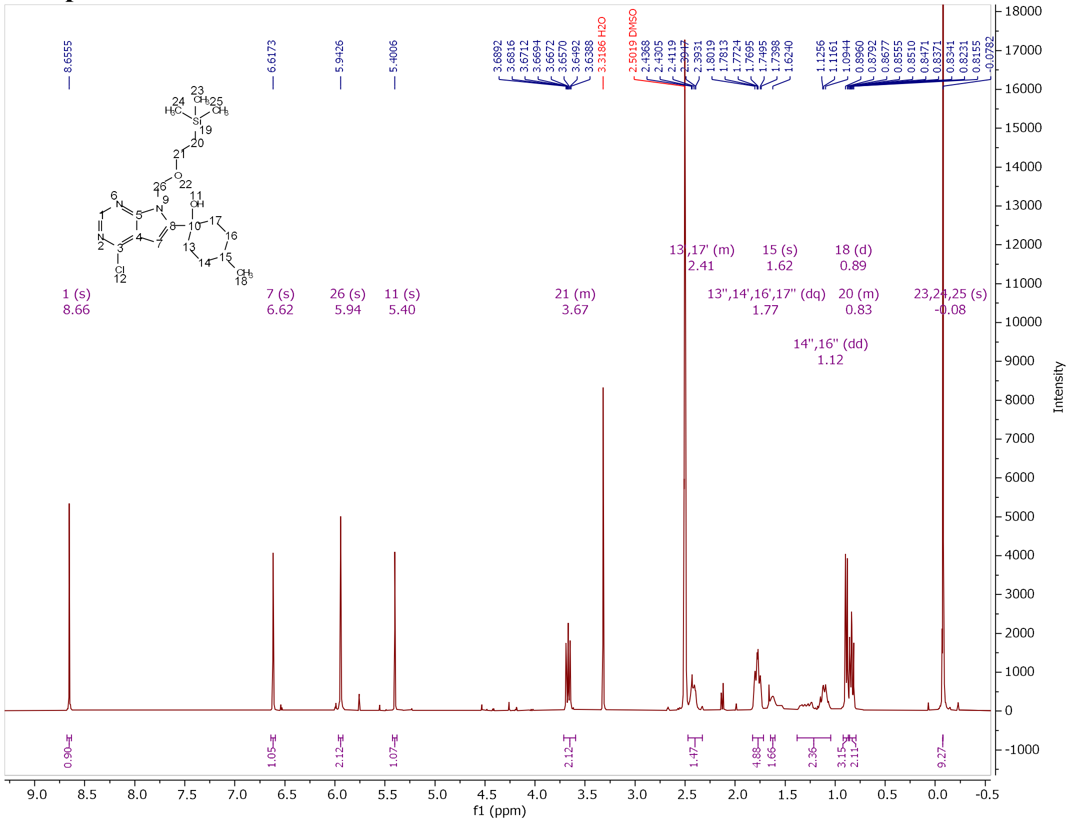

Figure S27. ^{1}H NMR (400 MHz, DMSO-d_6) spectrum of compound **3n**.

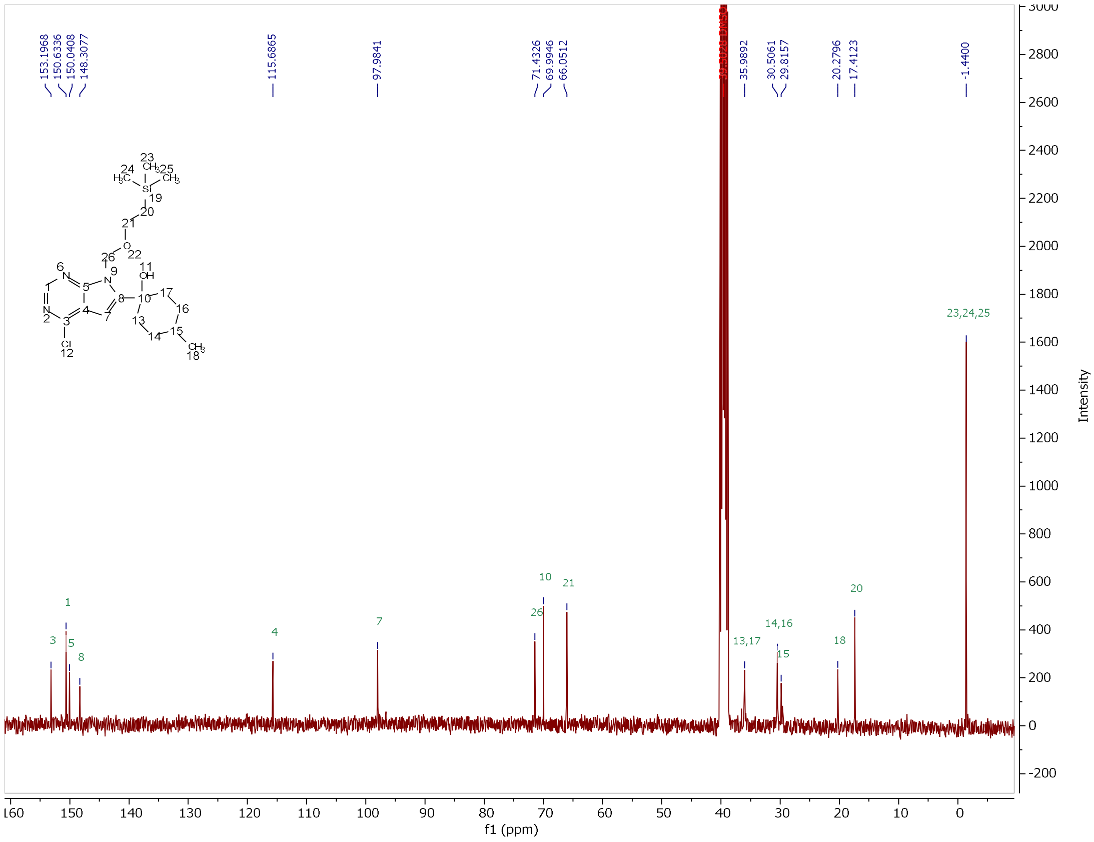

Figure S28. ^{13}C NMR (100 MHz, DMSO-d_6) spectrum of compound **3n**.

Compound 3o

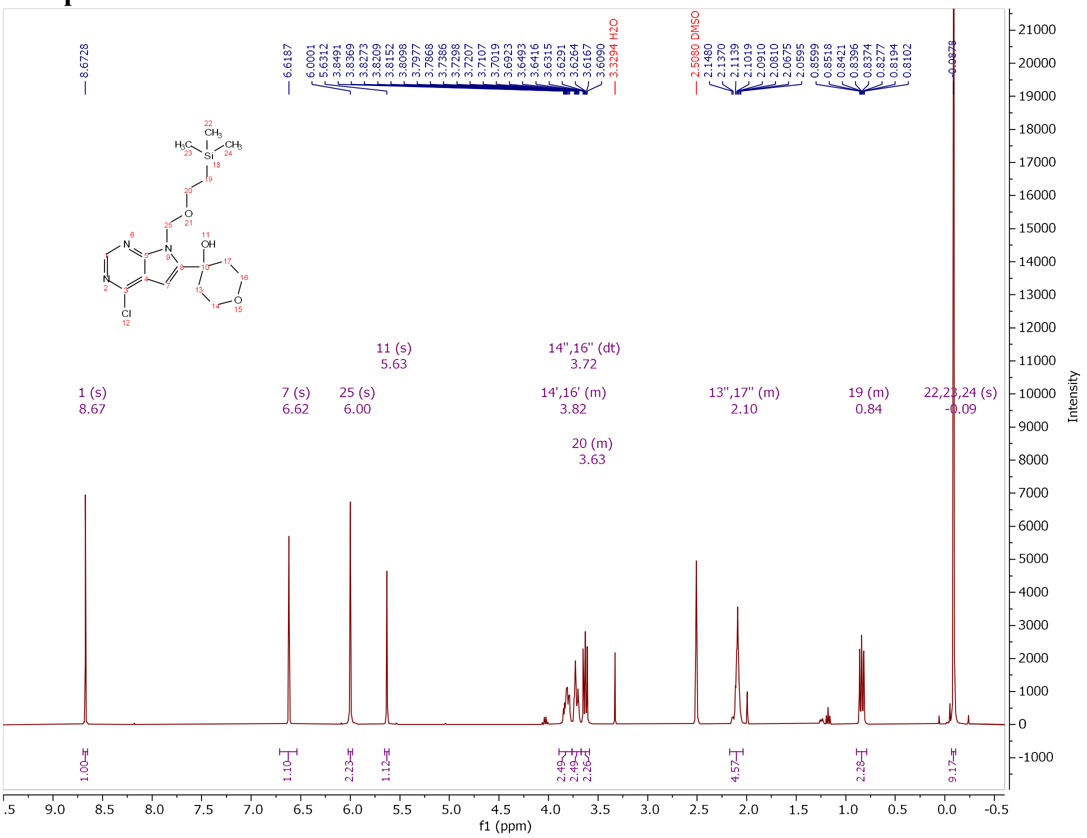

Figure S29. ^{1}H NMR (400 MHz, DMSO-d_6) spectrum of compound **3o**.

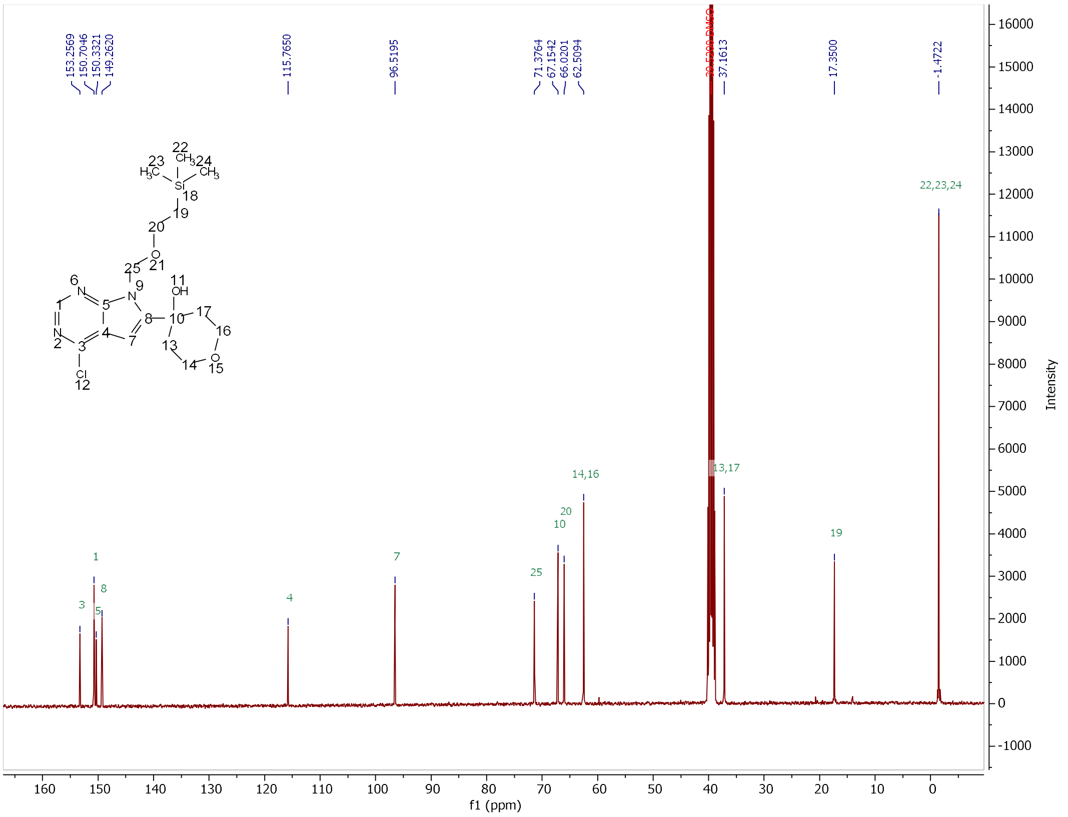

Figure S30. ^{13}C NMR (100 MHz, DMSO-d_6) spectrum of compound **3o**.

Compound 3p

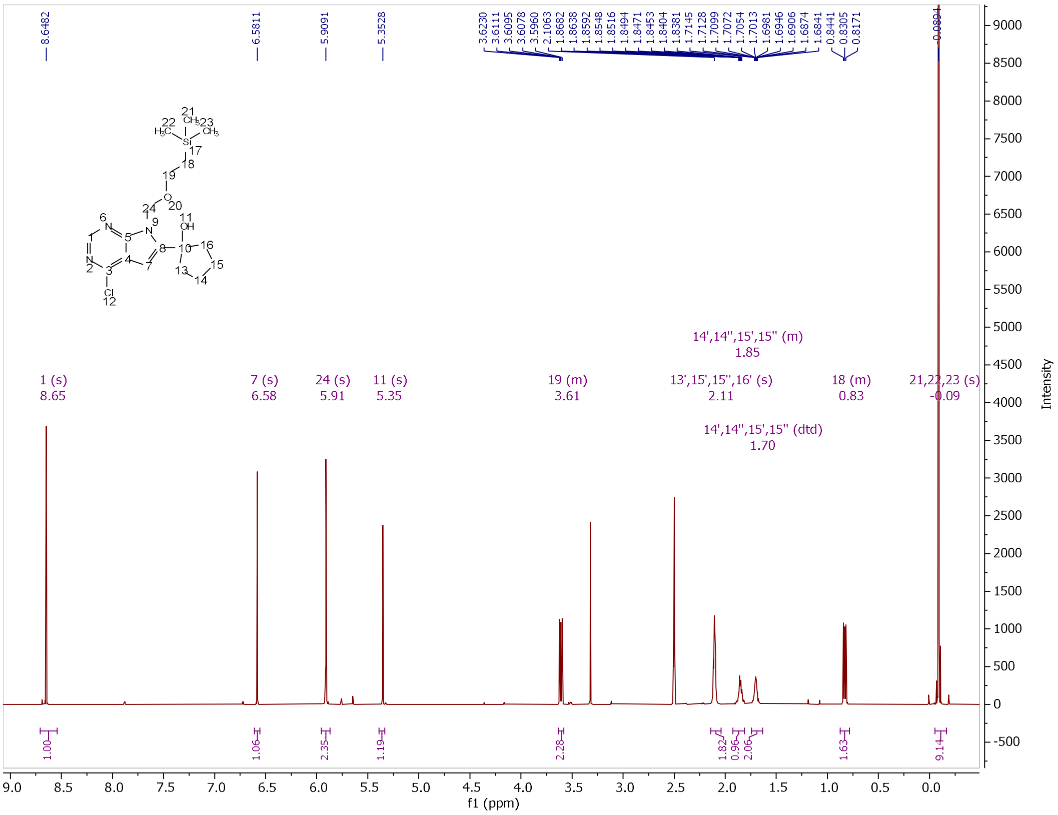

Figure S31. ^{1}H NMR (600 MHz, DMSO-d_6) spectrum of compound **3p**.

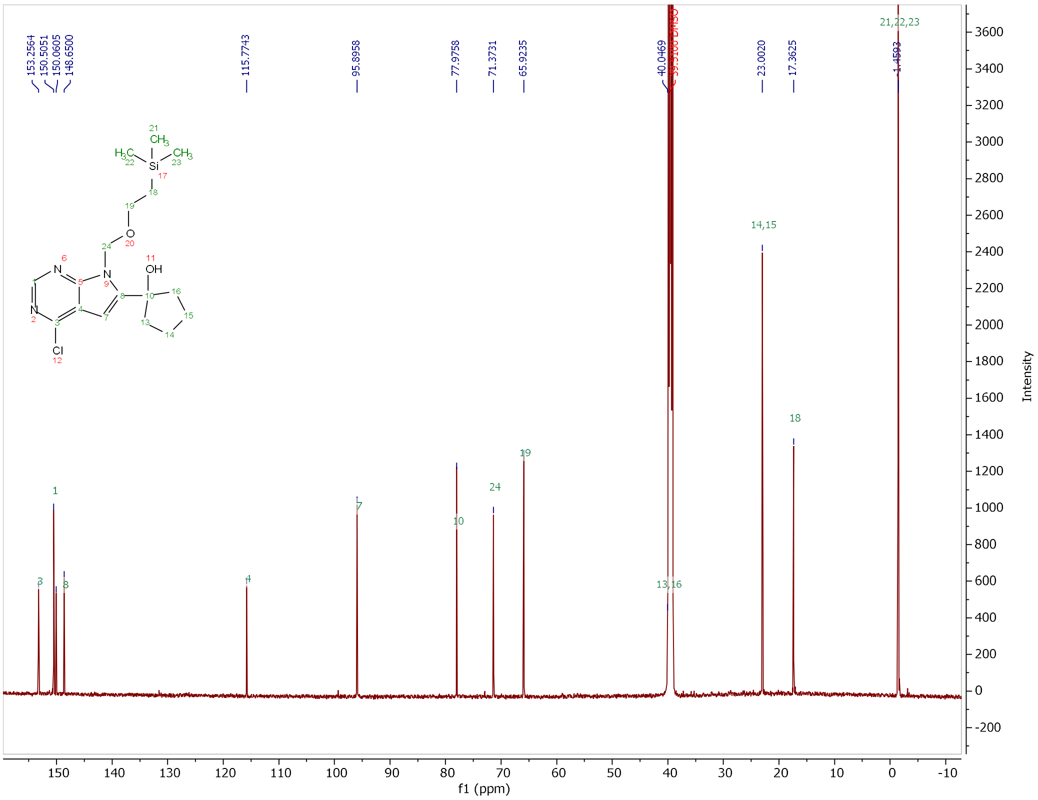

Figure S32. ^{13}C NMR (150 MHz, DMSO-d_6) spectrum of compound **3p**.

NMR spectra of compounds 1 and 4-8

Compound 1

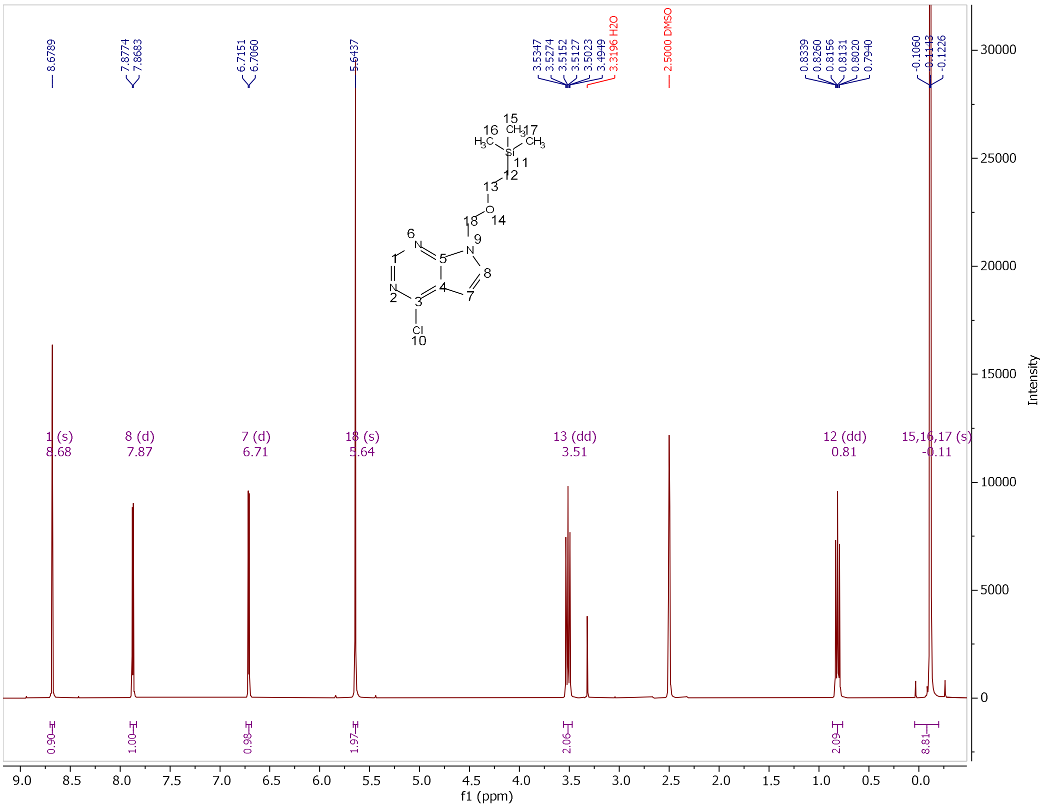

Figure S33. ^{1}H NMR (400 MHz, DMSO-d_6) spectrum of compound **1**.

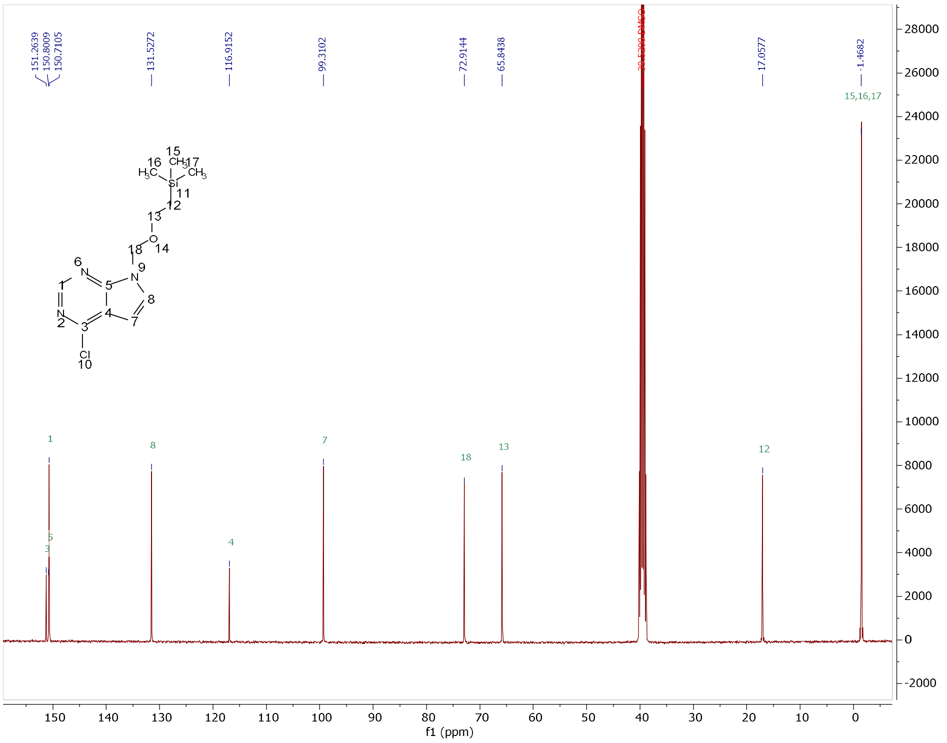

Figure S34. ^{13}C NMR (100 MHz, DMSO-d_6) spectrum of compound **1**.

Compound 4

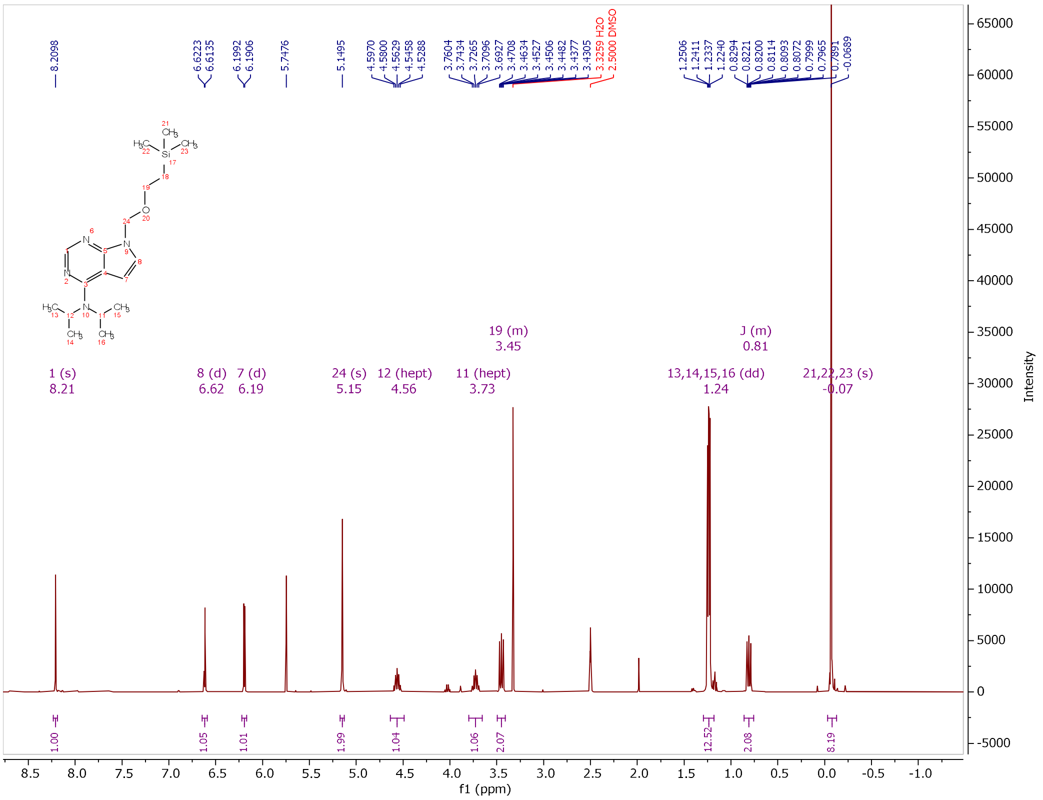

Figure S35. ^{1}H NMR (600 MHz, DMSO-d_6) spectrum of compound **4**.

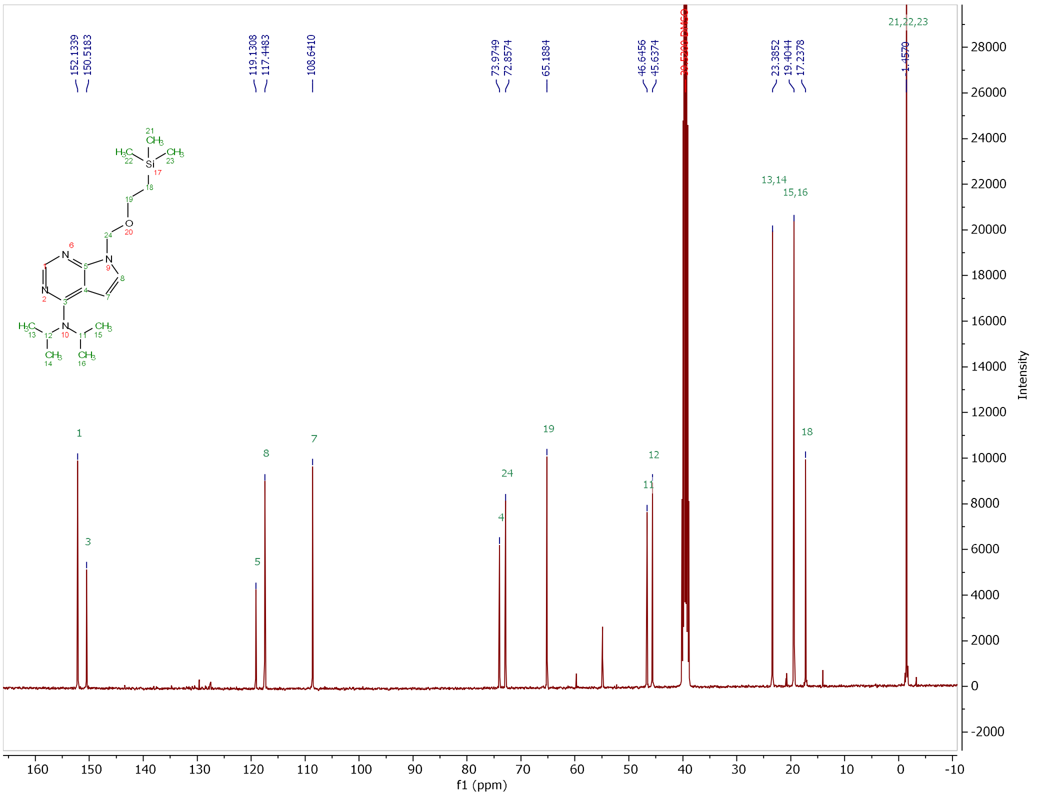

Figure S36. ^{13}C NMR (150 MHz, DMSO-d_6) spectrum of compound **4**.

Compound 5

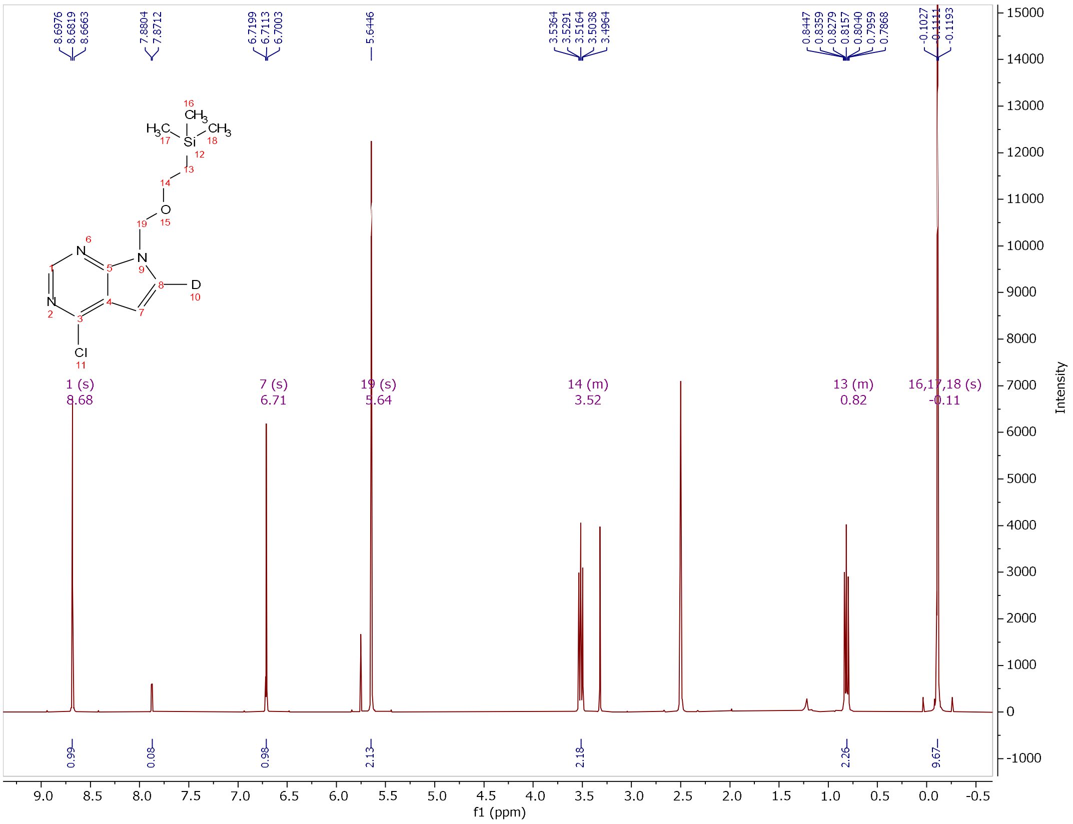

Figure S37. ^{1}H NMR (400 MHz, DMSO-d_6) spectrum of compound **5**.

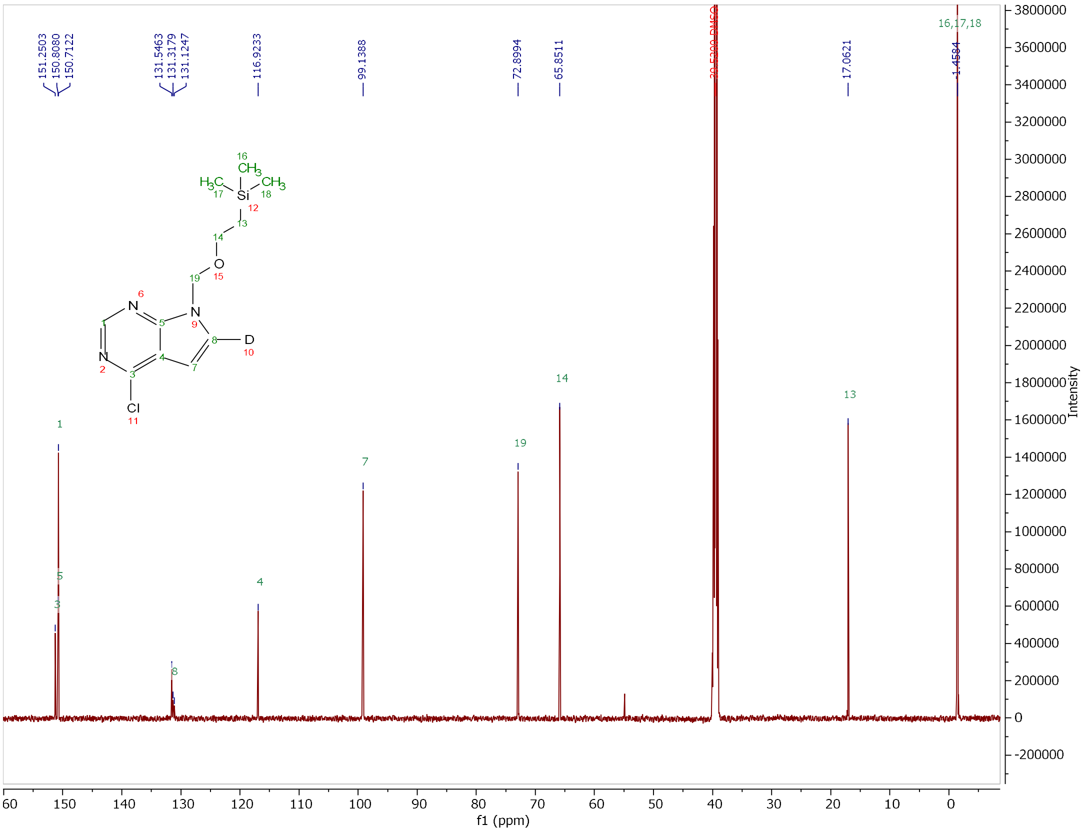

Figure S38. ^{13}C NMR (100 MHz, DMSO-d_6) spectrum of compound **5**.

Compound 6

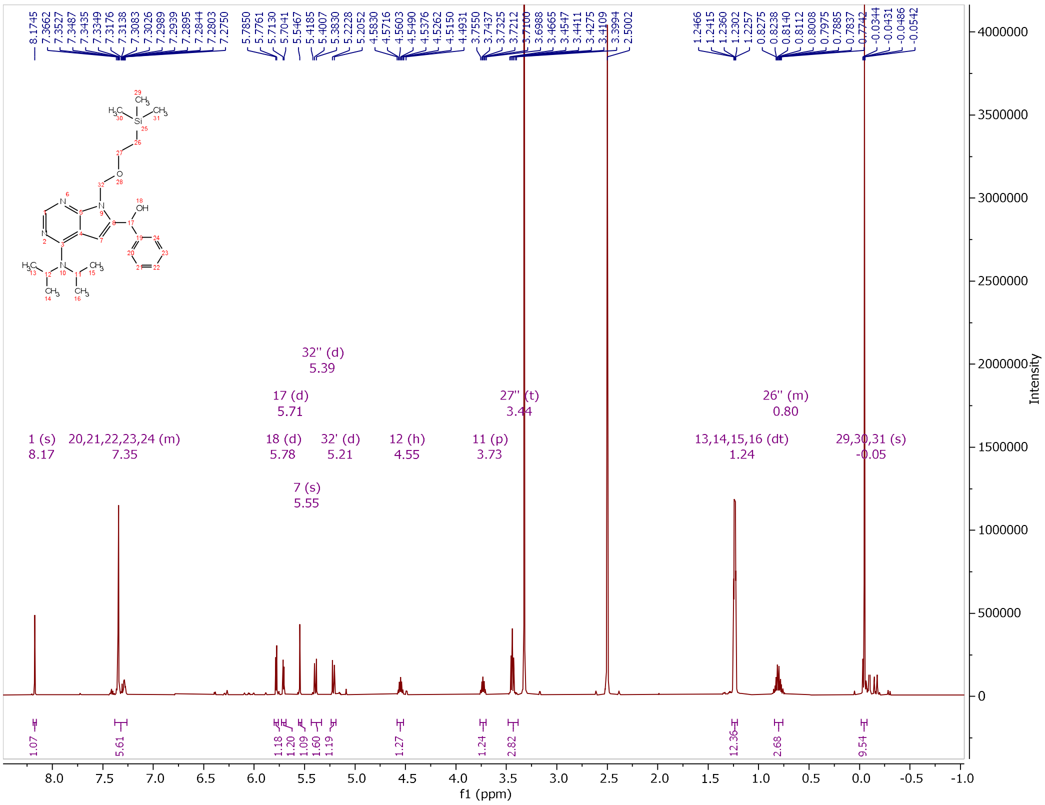

Figure S39. ^{1}H NMR (600 MHz, DMSO-d_6) spectrum of compound **6**.

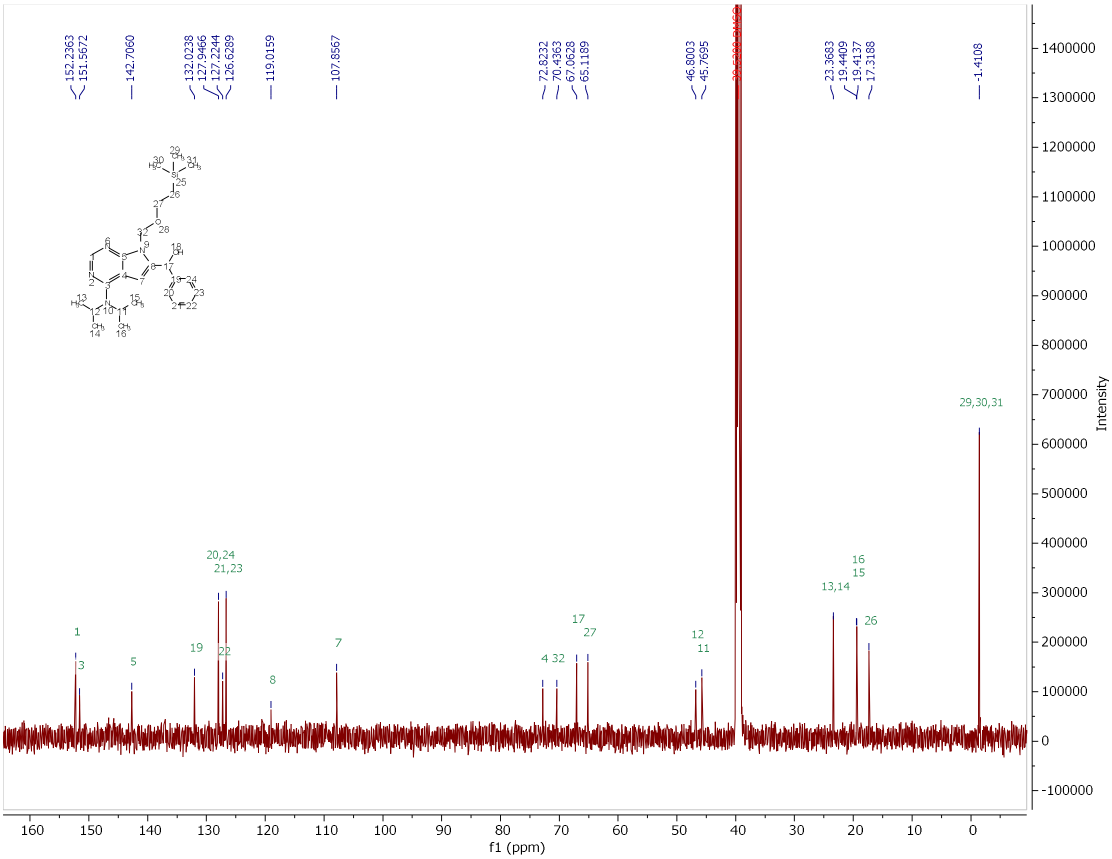

Figure S40. ^{13}C NMR (150 MHz, DMSO-d_6) spectrum of compound **6**.

Compound 8

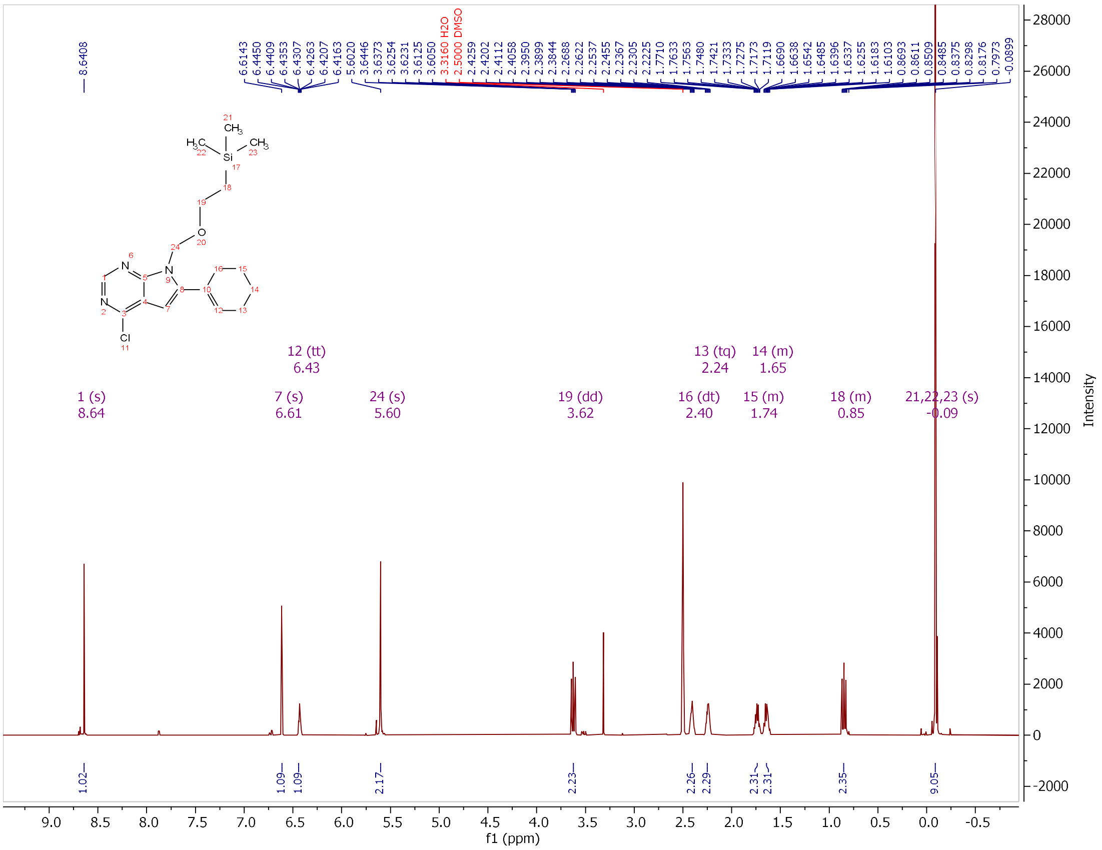

Figure S41. ^{1}H NMR (400 MHz, DMSO-d_6) spectrum of compound **8**.

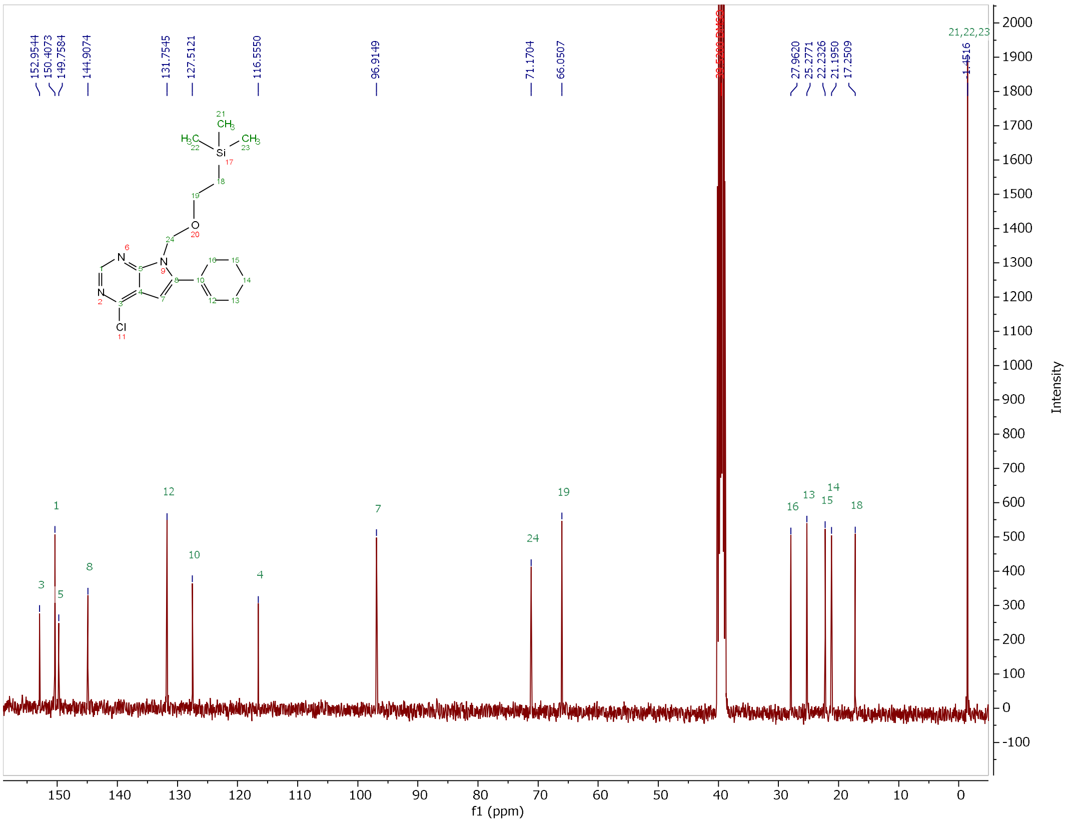

Figure S42. ^{13}C NMR (150 MHz, DMSO-d_6) spectrum of compound **8**.
